# The genetic architecture of the maize progenitor, teosinte, and how it was altered during maize domestication

**DOI:** 10.1371/journal.pgen.1008791

**Published:** 2020-05-14

**Authors:** Qiuyue Chen, Luis Fernando Samayoa, Chin Jian Yang, Peter J. Bradbury, Bode A. Olukolu, Michael A. Neumeyer, Maria Cinta Romay, Qi Sun, Anne Lorant, Edward S. Buckler, Jeffrey Ross-Ibarra, James B. Holland, John F. Doebley

**Affiliations:** 1 Laboratory of Genetics, University of Wisconsin–Madison, Madison, Wisconsin, United States of America; 2 Department of Crop Science, North Carolina State University, Raleigh, North Carolina, United States of America; 3 US Department of Agriculture–Agricultural Research Service, Cornell University, Ithaca, New York, United States of America; 4 Department of Entomology and Plant Pathology, University of Tennessee, Knoxville, Tennessee, United States of America; 5 Genomic Diversity Facility, Cornell University, Ithaca, New York, United States of America; 6 Department of Evolution and Ecology, University of California, Davis, California, United States of America; 7 US Department of Agriculture–Agricultural Research Service Plant Science Research Unit, North Carolina State University, Raleigh, North Carolina, United States of America; University of Georgia, UNITED STATES

## Abstract

The genetics of domestication has been extensively studied ever since the rediscovery of Mendel’s law of inheritance and much has been learned about the genetic control of trait differences between crops and their ancestors. Here, we ask how domestication has altered genetic architecture by comparing the genetic architecture of 18 domestication traits in maize and its ancestor teosinte using matched populations. We observed a strongly reduced number of QTL for domestication traits in maize relative to teosinte, which is consistent with the previously reported depletion of additive variance by selection during domestication. We also observed more dominance in maize than teosinte, likely a consequence of selective removal of additive variants. We observed that large effect QTL have low minor allele frequency (MAF) in both maize and teosinte. Regions of the genome that are strongly differentiated between teosinte and maize (high F_ST_) explain less quantitative variation in maize than teosinte, suggesting that, in these regions, allelic variants were brought to (or near) fixation during domestication. We also observed that genomic regions of high recombination explain a disproportionately large proportion of heritable variance both before and after domestication. Finally, we observed that about 75% of the additive variance in both teosinte and maize is “missing” in the sense that it cannot be ascribed to detectable QTL and only 25% of variance maps to specific QTL. This latter result suggests that morphological evolution during domestication is largely attributable to very large numbers of QTL of very small effect.

## Author summary

Although the genetics of trait differences between crops and their progenitors has been extensively studied, far less is known about the genetic architecture of trait variation in crop progenitors and how this architecture was altered during domestication. Here, we address this issue by comparing the genetic architecture of 18 domestication traits in maize and its ancestor teosinte using matched populations. Our results show that genetic architecture was reshaped during domestication in multiple ways. Maize has a greatly reduced number of QTL for domestication traits relative to teosinte and alleles at these QTL show greater dominance in maize. QTL alleles of large effect are present in both maize and teosinte, but more common in maize. We observed that regions of the genome that are strongly differentiated between teosinte and maize (high F_ST_) explain less additive variation in maize than teosinte and that genomic regions of high recombination explain a disproportionately large proportion of heritable variance both before and after domestication. Finally, we observed that about 75% of heritability is “missing” in the sense that it not associated with detectable QTL, which suggests that the raw material for domestication was largely composed of vast numbers of QTL of diminishingly small effects.

## Introduction

Ever since Charles Darwin employed domestication as a model for natural evolution [1], crop domestication has been extensively studied by evolutionary biologists [2–7]. Part of the power of domestication as a model for evolution is that the crop ancestors are extant and cross-compatible with crop species, allowing genetic dissection of the inheritance of domestication traits. Domestication also allows us to discern the impact of selection on genetic architecture through comparison of pre- and post-domestication populations. Since the genetic architecture of the ancestral species influences the possible outcomes when selection is applied to move a population to a new optimum, such comparisons can reveal how genetic architecture can both facilitate and constrain domestication [8].

Maize and teosinte offer an excellent system for genetic analysis of domestication. Both phylogenetic and archaeological evidence revealed that maize was domesticated from Balsas teosinte (*Z*. *mays* ssp. *parviglumis*) by a single domestication event in southern Mexico about 9,000 years ago [9, 10]. As outcrossing species, teosinte and maize conform to many of the assumptions underlying standard population genetic models, empowering the application of diverse evolutionary analyses [11]. Maize has undergone dramatic morphological change from its wild ancestor teosinte during domestication. A typical teosinte plant has multiple long lateral branches, each tipped with a tassel, whereas a typical maize plant has one or two short branches, each tipped with a single ear. A teosinte plant produces many two-ranked ears, each with only a few fruitcase-enveloped kernels, easily shattering into single-seed units at maturity. In contrast, a maize plant produces only one or two multiple-ranked ears, each with hundreds of naked grains, remaining intact on the cob at maturity. Finally, maize has a large number of genomic and genetic resources available (https://maizegdb.org/), including high-quality genomes of inbred lines [12–15], high-density genotypic data [16], and diverse mapping populations [17, 18].

In this paper, we report a comprehensive comparison of genetic architecture in 18 domestication traits for matched populations of a maize landrace and teosinte. Our overarching goal is to better define genetic architecture in maize and teosinte and ask how it changed as a result of domestication. From our analyses, we observed that genetic architecture has been reshaped during domestication with a substantial reduction in the number of segregating QTL affecting domestication traits. We observed that regions of the genome that are strongly divergent between teosinte and maize (high F_ST_) explain less variation in maize than teosinte. We also observed that genomic regions of high recombination explain a disproportionately large proportion of heritable variance both before and after domestication. Finally, we observed that only 25% of the additive variance in both teosinte and maize can be ascribed to specific QTL. Overall, our work suggests that trait evolution during maize domestication is largely attributable to very large numbers of QTL of very small effects.

## Results

### QTL and their effects

#### Our maize landrace population shows a strongly reduced number of QTL for domestication traits relative to our teosinte population, indicating that the domestication bottleneck and/or selection caused the loss or fixation of many functional alleles

**Fig 1:**
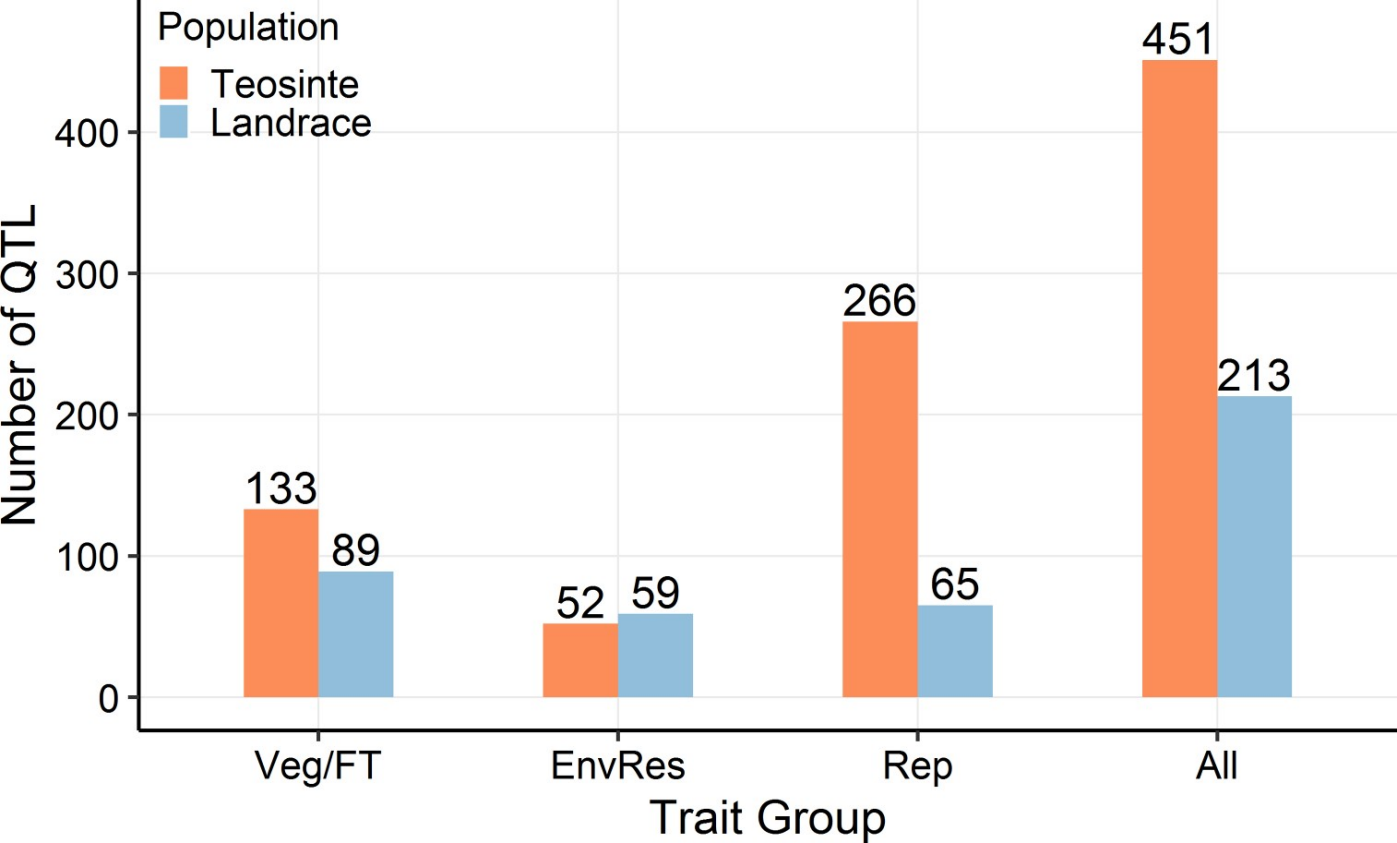
QTL summary. The number of QTL is plotted against trait groups for teosinte and maize landrace, respectively. The exact number of QTL in each group is shown above each bar. The trait groups are abbreviated as Veg/FT for Vegetative/Flowering Time, EnvRes for Environmental Response, and Rep for Reproductive.

Using a stepwise regression approach, we performed genome-wide QTL scans for 18 domestication traits scored in both our teosinte and maize landrace populations (Table 1). We detected a total of 451 QTL in teosinte (ranging from 3 to 52 per trait) but only 213 QTL in maize landrace (ranging from 0 to 27) (Fig 1; S1 Table). We grouped the 18 traits into three previously defined groups: vegetative/flowering time, reproductive, and environmental response traits [8]. Among the three predefined trait groups, reproductive traits showed the greatest reduction in the number of QTL, and vegetative traits a strong, but more modest reduction. Environmental response traits showed slightly more QTL in maize landrace than in teosinte, but the overall trend is a substantial reduction in QTL numbers after domestication. The most striking difference was observed for GW, for which we detected 52 QTL in teosinte but only 6 QTL in maize landrace (S1 Table).

**Table 1:** Trait abbreviations.

Trait	Acronym	Units	Trait Group
Days to Anthesis	DTA	days	Veg/FT
Days to Silking	DTS	days	Veg/FT
Plant Height	PLHT	cm	Veg/FT
Leaf Length	LFLN	cm	Veg/FT
Leaf Width	LFWD	cm	Veg/FT
Tiller Number	TILN	count	EnvRes
Prolificacy	PROL	count	EnvRes
Lateral Branch Node Number	LBNN	count	EnvRes
Lateral Branch Length	LBLN	mm	EnvRes
Lateral Branch Internode Length	LBIL	mm	EnvRes
Ear Length	EL	mm	Rep
Cupules per Row	CUPR	count	Rep
Ear Diameter	ED	mm	Rep
Grains Per Ear	GE	count	Rep
Ear Internode Length	EILN	mm	Rep
Total Grain per Plant	TGPP	count	Rep
Total Grain Weight per Plant	TGWP	g	Rep
Grain Weight	GW	mg	Rep

List of 18 teosinte-maize landrace comparable traits and the corresponding acronyms, units and trait groups. Veg/FT: Vegetative/Flowering Time; EnvRes: Environmental Response; Rep: Reproductive.

#### Large effect QTL were observed in both populations but appear more common in maize landrace

**Fig 2:**
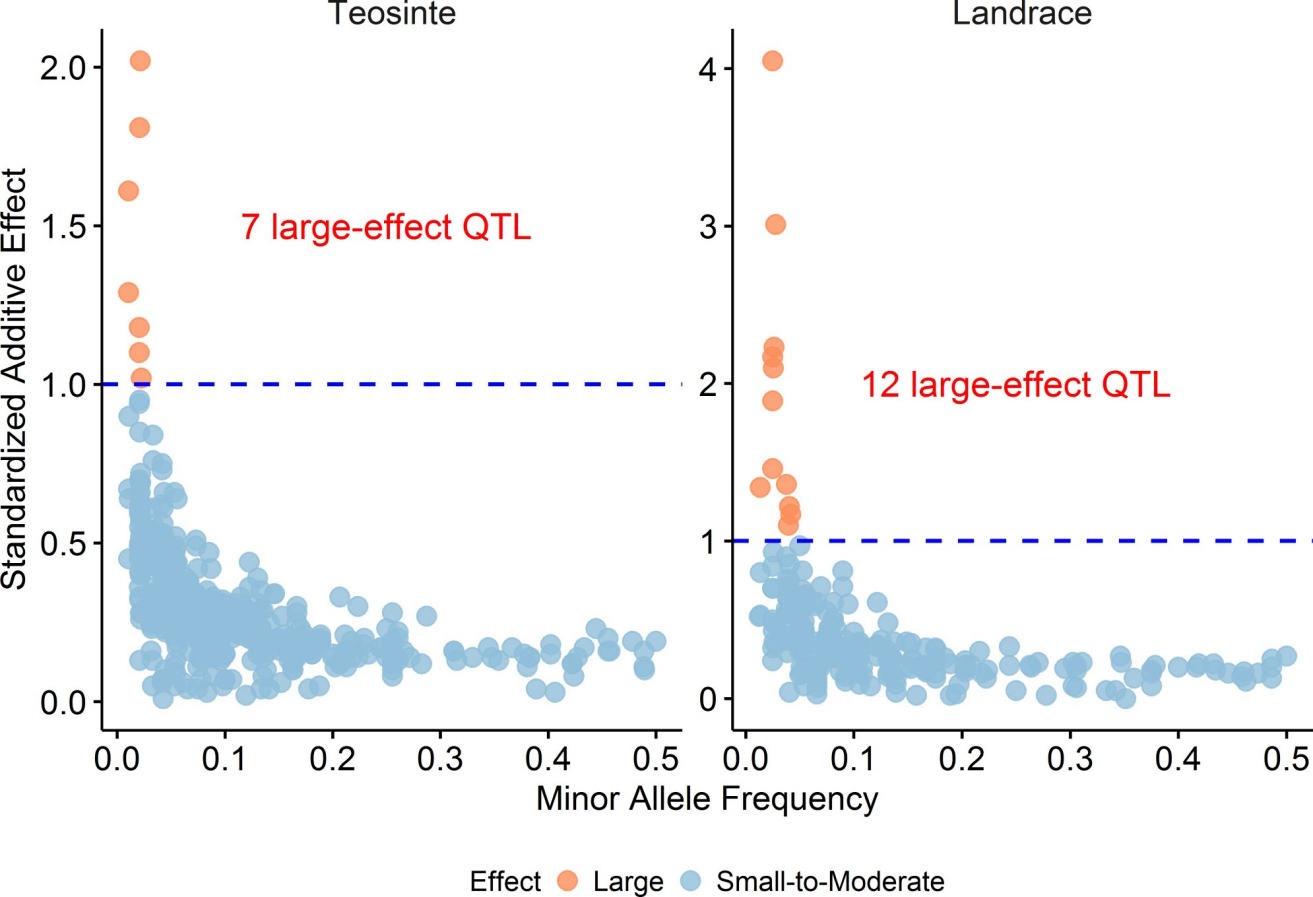
The relationship between effect size and minor allele frequency (MAF). The standardized additive effect, calculated as additive effect by phenotypic standard deviation in absolute value, is plotted against MAF for each QTL. MAF was calculated from parent data. Large effect QTL is defined as a QTL with a standardized additive effect greater than 1 phenotypic standard deviation as indicated by blue dotted lines.

**Fig 3:**
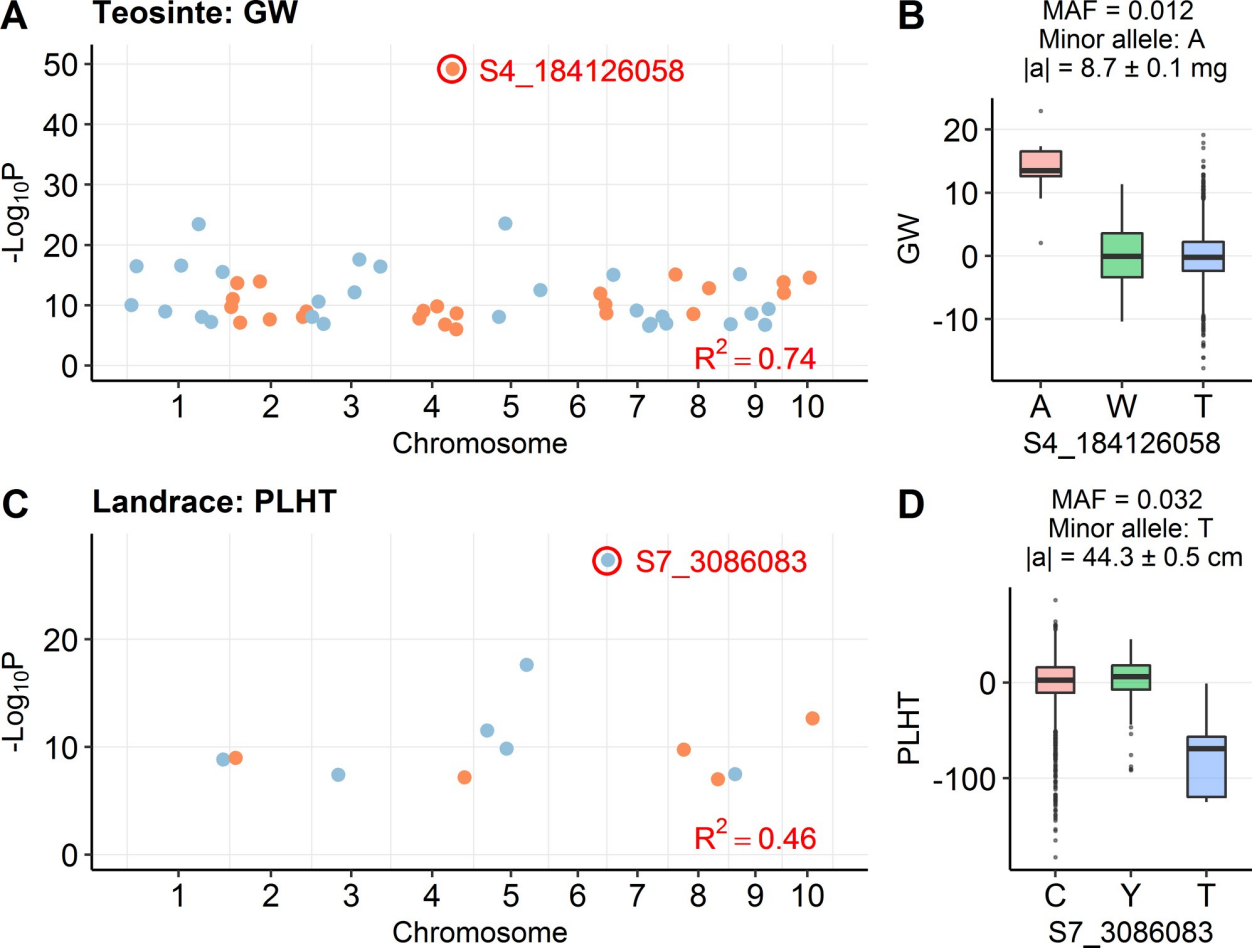
Examples of QTL under mutation-selection balance. (A) The distribution of detected QTL for GW in teosinte. The most significant QTL is labeled as S4_184126058. (B) The phenotypic effect of QTL S4_184126058. (C) The distribution of detected QTL for PLHT in maize landrace. The most significant QTL is labeled as S7_3086083. (D) The phenotypic effect of QTL S7_3086083. In (A) and (C), QTL in different chromosomes are shown in different colors; R^2^ stands for r-square explained by overall QTL model. In (B) and (D), the phenotype is shown in residuals after accounting for covariates; |a| stands for absolute additive effect (mean ± s.e.).

Here, we define large effect as a QTL with a standardized additive effect greater than 1 phenotypic standard deviation, where the additive effect was estimated by stepwise regression. Seven of the 451 QTL in teosinte have a large effect and 12 of the 213 QTL in maize landrace have a large effect (Fig 2; S1 Table). The frequency of 5.6% in maize landrace is significantly larger than 1.6% in teosinte (P = 0.0052, two-sided Fisher's exact test). Thus, large effect QTL occur in both populations but they are more common (both in frequency and total number) in maize landrace. One example of a large effect QTL is a GW QTL on chromosome 4 in teosinte (T/A, S4_184126058, P = 6.38E-50) (Fig 3A), where this QTL has an additive effect of 8.7 mg in GW, which corresponds to a 34% increase from the population mean of 25.6 mg or 1.61 phenotypic standard deviations. Another example is a major effect QTL for PLHT on chromosome 7 in maize landrace (C/T, S7_3086083, P = 4.10E-28) (Fig 3B), where this QTL has an additive effect of 44.3 cm in PLHT, which corresponds to a 19% decrease from the population mean of 230.5 cm or 1.34 phenotypic standard deviations.

#### Overall, large effect QTL have low MAF for both teosinte and maize landrace, suggesting mutation-selection balance may play an important role in modulating genetic variation in both taxa

By plotting the distribution of standardized additive effect against MAF for all QTL (Fig 2), we observed that the largest effect QTL have low MAF in both teosinte and maize landrace. The rarest alleles (parent MAF < 0.05) have effects that are in a range from 0.01 to 4.05 phenotypic standard deviations, while the effects of the most common alleles (parent MAF > 0.3) never exceed 0.3 phenotypic standard deviations. This is true of both teosinte and maize landrace. For example, the largest effect QTL for GW in teosinte (T/A, S4_184126058, P = 6.38E-50) has a MAF of 0.012 and the minor allele comes from a single one of our 49 teosinte parents (PC_N57_ID2) (Fig 3A). Similarly, the largest effect QTL for PLHT in maize landrace (C/T, S7_3086083, P = 4.10E-28) has a MAF of 0.032 and the minor allele comes from a single one of our 40 maize landrace parents (164_1) (Fig 3B). These observations suggest that most segregating large effect alleles are likely the product of relatively recent mutation and selection has not yet had sufficient time to either remove them from the population or bring them to fixation.

A concern with this analysis is that effect estimates for rare alleles may be biased upwards because of the relatively small sample size for rare alleles which may cause these QTL to only be detected when their effects are overestimated. To address this concern, we plotted the distribution of standardized additive effect for SNPs whether the SNP is significant or not by each trait, where the additive effects were estimated from our GLM model. Also, to minimize error in the estimation of the effects of rare allele, we required a minimum of 10 homozygous plants for the rare allele. Examination of the plot of MAF by effect size this way still indicates that rare alleles have larger effects for all 18 traits (S1 Fig). This result indicates that bias in the estimate of rare allele effect sizes cannot fully explain the observed relationship between effect size and MAF.

#### Balancing selection may maintain some genetic variation in both teosinte and maize landrace

**Fig 4:**
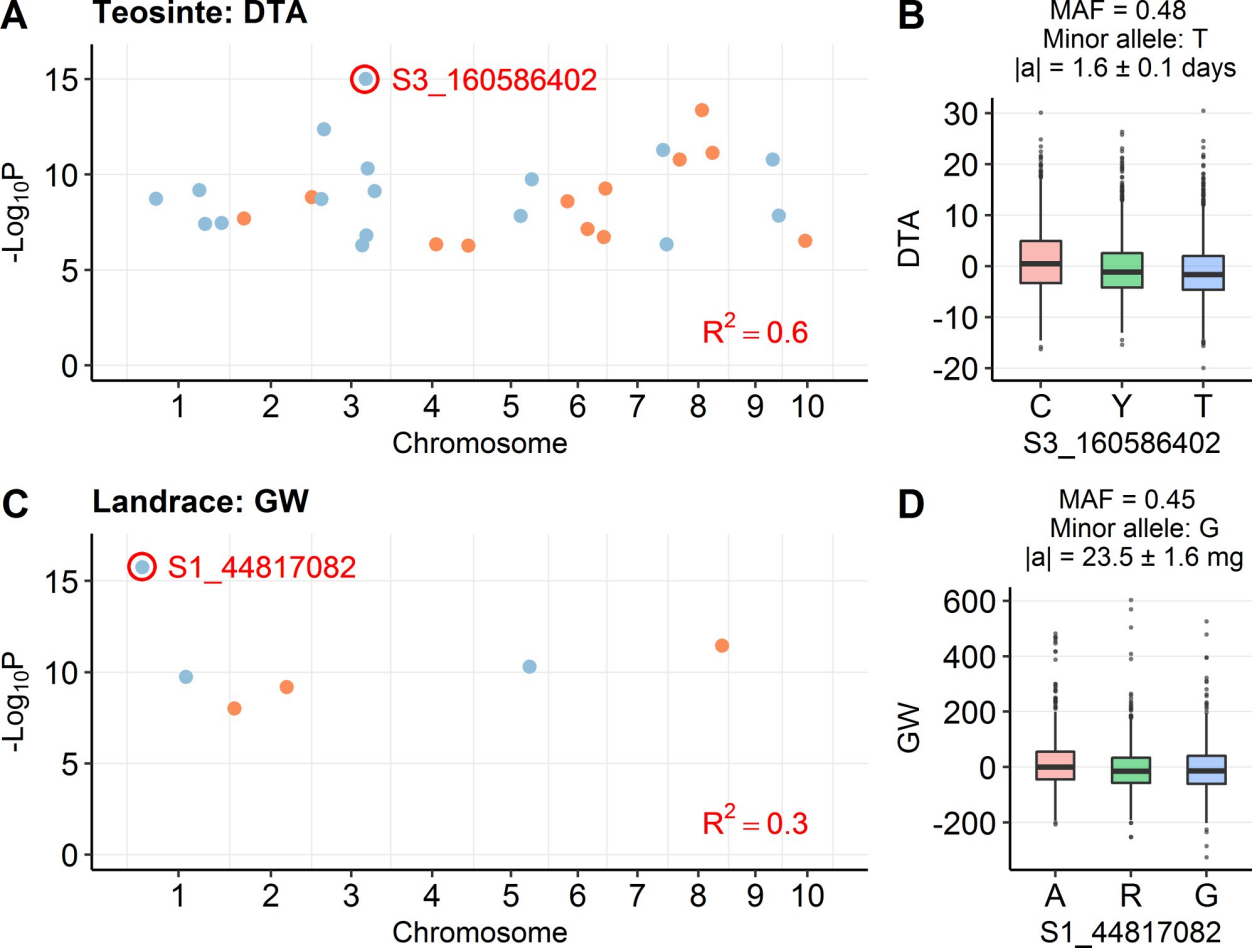
Examples of QTL under balancing selection. (A) The distribution of detected QTL for DTA in teosinte. The most significant QTL is labeled as S3_160586402. (B) The phenotypic effect of QTL S3_160586402. (C) The distribution of detected QTL for GW in maize landrace. The most significant QTL is labeled as S1_44817082. (D) The phenotypic effect of QTL S1_44817082. In (A) and (C), QTL in different chromosomes are shown in different colors; R^2^ stands for r-square explained by overall QTL model. In (B) and (D), the phenotype is shown in residuals after accounting for covariates; |a| stands for absolute additive effect (mean ± s.e.).

Examination of the plots of MAF by effect size shows a few QTL with high frequency and moderately strong effects (Fig 2). Such alleles could be maintained in the population by balancing selection. An interesting example of this is a QTL on chromosome 3 for DTA in teosinte (C/T, S3_160586402, P = 9.64E-16), where the MAF is 0.48 and the additive effect is 0.19 phenotypic standard deviations (Fig 4A). This pattern fits the expectation of balancing selection. Similarly, we observed a QTL on chromosome 1 for GW in maize landrace (A/G, S1_44817082, P = 1.75E-16), where the MAF is 0.45 and the additive effect is 0.25 phenotypic standard deviations (Fig 4B), suggesting this QTL may be under balancing selection.

#### There is more dominance in maize landrace than teosinte, which may be related to the depletion of additive variance by selection during domestication

**Fig 5:**
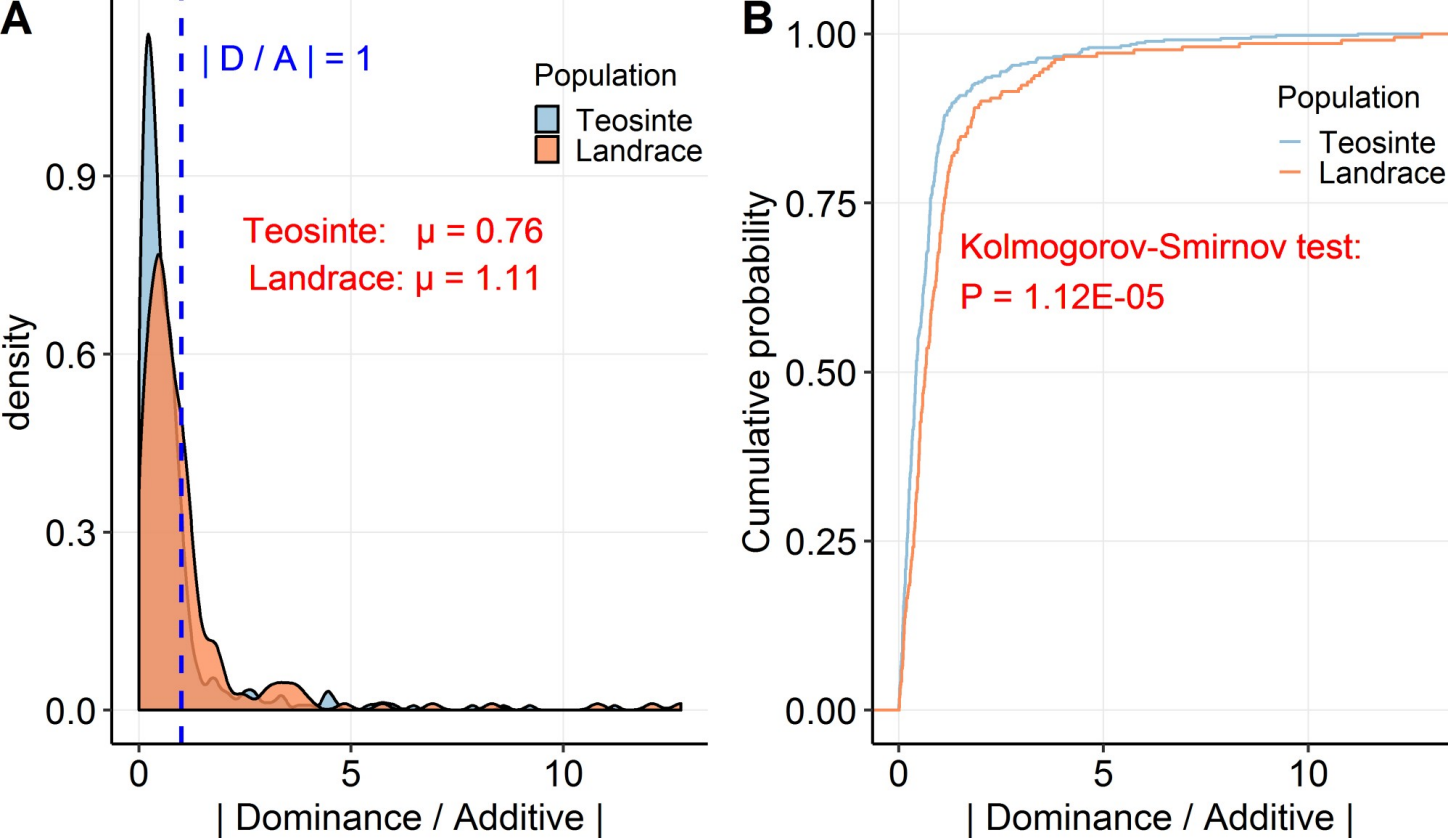
The distribution of dominance-by-additive effect. (A) Density is plotted for the ratio of dominance by additive effects in absolute value. The blue dotted line indicates |D/A| = 1 and the mean value (μ) of |D/A| for teosinte and maize landrace population is shown in red text. (B) The cumulative distribution is plotted for |D/A|, with P value calculated by Kolmogorov-Smirnov test to reflect the difference in the two distributions.

Previously, Yang et al. [8] reported that there is more dominance genetic variance than additive variance in maize landrace relative to teosinte, and these authors suggested that this pattern may be the result of selection during domestication having depleted the additive variance. Here, by plotting the ratio of dominance to additive effects (D/A) (Fig 5A), we observed a small shift to greater dominance in maize landrace than in teosinte. The mean absolute value of the D/A ratio is 0.76 in teosinte (ranging from 0.001 to 11.21), while the mean absolute value of the D/A ratio is 1.11 in maize landrace (ranging from 0.01 to 12.77). Moreover, the cumulative distribution plot of D/A shows a significant difference in the two distributions (Fig 5B; P = 1.12E-05, Kolmogorov-Smirnov test). The increase of the D/A ratio in maize landrace relative to teosinte suggests that additive variants were more likely fixed or lost during domestication and thus aligns with the observation of Yang et al. [8].

### Candidate genes for QTL in teosinte and maize landrace

#### Our analyses identify multiple strong candidate genes in both teosinte and maize landrace

Although our population design and stepwise regression analysis provide only limited precision for mapping QTL to the underlying genes, we saw multiple well-defined peaks in the Manhattan plots from our GLM scan of the genome, allowing us to identify candidate genes associated with the most significant QTL peaks (P<1E-20) (Table 2). There were 9 such QTL in teosinte and 7 QTL in maize landrace. In teosinte, potential candidate genes were defined for DTS, PLHT, LFWD, PROL, ED and GW. For example, the largest effect QTL for DTS is located on chromosome 3 (G/A, S3_161034847, P = 4.87E-40) upstream of *SBP-transcription factor 22* and a known flowering time gene *MADS-transcription factor 69* (Zm*MADS69*, *Zm00001d042315*). Interestingly, the largest QTL for DTA (C/T, S3_160586402, P = 9.64E-16) is nearby, located downstream of Zm*MADS69*. For GW, the largest effect QTL is located on chromosome 4 (T/A, S4_184126058, P = 6.38E-50) upstream of AP2-EREBP-transcription factor 17. A second interesting candidate is the next gene downstream, *trehalose-6-phosphate phosphatase 4* (*TPP4*, *Zm00001d052227*). Interestingly, this QTL is located within the inversion on chromosome 4 (*Inv4m*, B73 RefGen v4: 171.8–186.0 Mb) [19]. For LFWD, the largest effect QTL is located on chromosome 6 (A/C, S6_170102149, P = 1.73E-25) in a gene encoding 2-oxoglutarate-dependent dioxygenase AOP1, but there is another gene nearby, *tassel sheath1* (*tsh1*, *Zm00001d039113*), known to effect leaf size. For PLHT, the largest effect QTL is located on chromosome 1 (C/T, S1_156526333, P = 6.45E-28) upstream of a gene encoding U-box domain-containing protein 13. There is another gene nearby, *rough sheath2* (*rs2*, *Zm00001d030737*), which encodes a MYB-domain protein and is expressed in lateral organ primordia and their initials. This gene has been reported to affect plant architecture including plant height [20].

**Table 2:** Candidate genes under P<1E-20.

Population	Trait	QTL	Chr	Position	Allele	Maf	probF	StdAddEffect	Candidate gene	Description
Teosinte	DTS	S3_161034847	3	161,034,847	G/A	0.14	4.87E-40	0.44	*ZmMADS69*	MADS-box transcription factor69
Teosinte	PLHT	S1_156526333	1	156,526,333	C/T	0.02	6.45E-28	1.10	*rs2*	rough sheath2
Teosinte	LFWD	S1_160665121	1	160,665,121	C/T	0.04	7.16E-25	0.84	*bsd2*	bundle sheath defective2
Teosinte	LFWD	S6_170102149	6	170,102,149	A/C	0.02	1.73E-25	0.95	*tsh1*	tassel sheath1
Teosinte	PROL	S1_194694757	1	194,694,757	C/T	0.02	5.43E-23	1.18		
Teosinte	ED	S4_182939860	4	182,939,860	C/T	0.01	9.70E-28	1.29	*TPP4*	trehalose-6-phosphate phosphatase4
Teosinte	GW	S1_213592730	1	213,592,730	G/A	0.19	3.53E-24	0.21		
Teosinte	GW	S4_184126058	4	184,126,058	T/A	0.01	6.38E-50	1.61	*TPP4*	trehalose-6-phosphate phosphatase4
Teosinte	GW	S5_94468458	5	94,468,458	C/T	0.14	2.73E-24	0.29		
Landrace	PLHT	S7_3086083	7	3,086,083	C/T	0.03	4.10E-28	1.34	*rs1*	rough sheath1
Landrace	TILN	S1_184111630	1	184,111,630	G/T	0.02	1.64E-21	0.64		
Landrace	TILN	S2_5692647	2	5,692,647	C/T	0.03	1.17E-23	0.34		
Landrace	TILN	S5_172000041	5	172,000,041	C/A	0.04	8.96E-31	0.61		
Landrace	TILN	S7_5797272	7	5,797,272	G/A	0.02	4.07E-25	0.80		
Landrace	EILN	S8_160608498	8	160,608,498	A/C	0.03	1.84E-25	1.46		
Landrace	TGWP	S1_247411382	1	247,411,382	G/A	0.04	3.46E-29	4.05		

In maize landrace, such large effect QTL were detected for PLHT, TILN, EILN and TGWP. For example, the largest effect QTL for PLHT is located on chromosome 7 (C/T, S7_3086083, P = 4.10E-28) just upstream of a gene encoding phosphoprotein phosphatase inhibitors. As was also true for the major PLHT QTL in teosinte, there is a *rough sheath1* gene (*rs1*, *Zm00001d018742*) nearby [21]. For EILN, the largest effect QTL is located on chromosome 8 (A/C, S8_160608498, P = 1.84E-25) upstream of a gene encoding SUPPRESSOR OF ABI3-5. For TILN, the QTL on chromosome 2 (C/T, S2_5692647, P = 1.17E-23) is located upstream of a gene encoding a zinc finger domain-containing protein.

### Teosinte and maize landrace have a similar level of missing heritability

The variation in our populations can be attributed to both QTL of effect sizes that are large enough to be detected by our QTL scan, as well as very small effect QTL that escape detection but still contribute to heritable variation. The latter control the “missing heritability”. By fitting linear models with QTL and/or covariates, we obtained the R^2^ values for the QTL alone, family effects (Principal Components, PCs) alone, and R^2^ values for variance explained by neither QTL nor family effects. We defined “missing heritability” as the portion of the additive variation not explained by the QTL. We compared these values to the additive genetic variance of each trait reported by Yang et al. [8].

#### Most heritable variation is "missing" in both teosinte and maize landrace, suggesting that undetectable small effect QTL predominate in the genetic architecture of these populations

**Fig 6:**
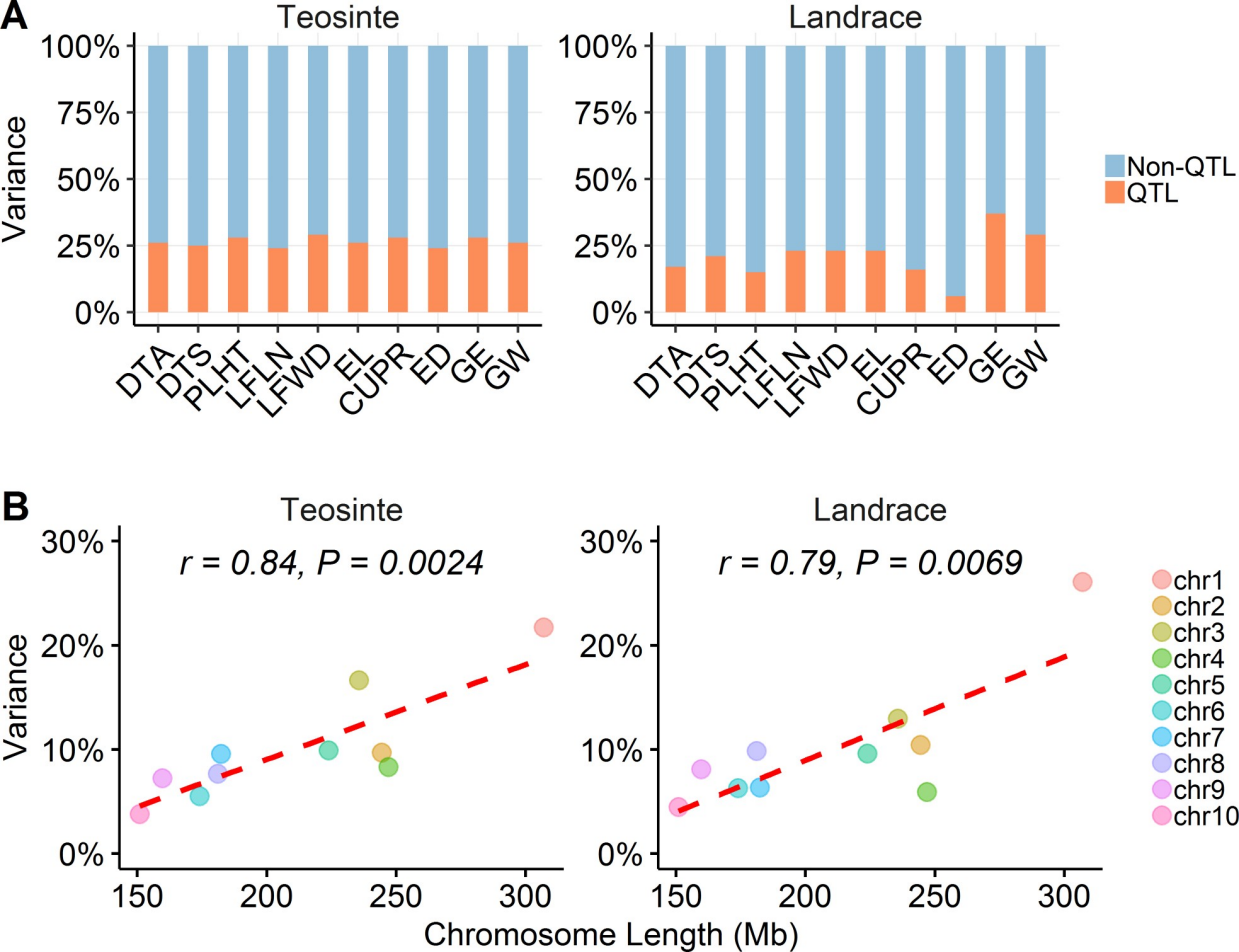
Missing heritability. (A) The percentage of variance explained by QTL and non-QTL, respectively. Most heritable variation is "missing" in both teosinte and maize landrace. (B) The relationship between chromosome length and the proportion of variance. The correlation coefficient *r* and corresponding *P* value is indicated. The proportion of variance explained by chromosomes is positively correlated with chromosome length in both teosinte and maize landrace.

We observed that detectable QTL could explain only 26% and 21% of additive genetic variance for teosinte and maize landrace, respectively, signifying that the remaining variation (74% and 79%, respectively) is missing (Fig 6A; S2 Table). This result indicates that QTL of very small effect explain most of the variance in both teosinte and maize landrace and that domestication did not alter the amount of missing heritability appreciably. Part of the missing heritability is captured by family effects (PCs in our stepwise regression model), corresponding to 46% for teosinte and 51% for maize landrace. These percentages may represent functional alleles that are unique to individual parents (rare alleles). Finally, about 17% and 26% of additive genetic variance in teosinte and maize landrace, respectively, cannot be associated with either QTL or family effects and may represent very small effect common alleles that occur in multiple families in our populations.

If alleles of very small effects distributed evenly throughout the genome are predominant in controlling heritable variation, then we should observe a correlation between chromosome length and the proportion of the variance explained. We performed variance component analysis (VCA) for 18 traits by chromosomes. The results show that the proportion of variance explained by chromosome is highly correlated with the length of chromosome for both teosinte (*r* = 0.84, *P* = 0.0024) and maize landrace (*r* = 0.79, *P* = 0.0069) (Fig 6B), a result consistent with an important role for small effect alleles explaining most heritable variance.

### Partitioning of the genetic variance by genomic features

To estimate the proportion of trait genetic variance associated with different features of the genome, we performed variance component analysis for 18 traits in both teosinte and maize landrace. This analysis involved classifying SNPs into subsets depending upon different genomic features, generating kinship matrices for each subset, and fitting these kinship matrices along with the phenotypic data to partition the genetic variance (heritability, h^2^).

#### High recombination regions explain most heritable variance in both teosinte and maize landrace

**Fig 7:**
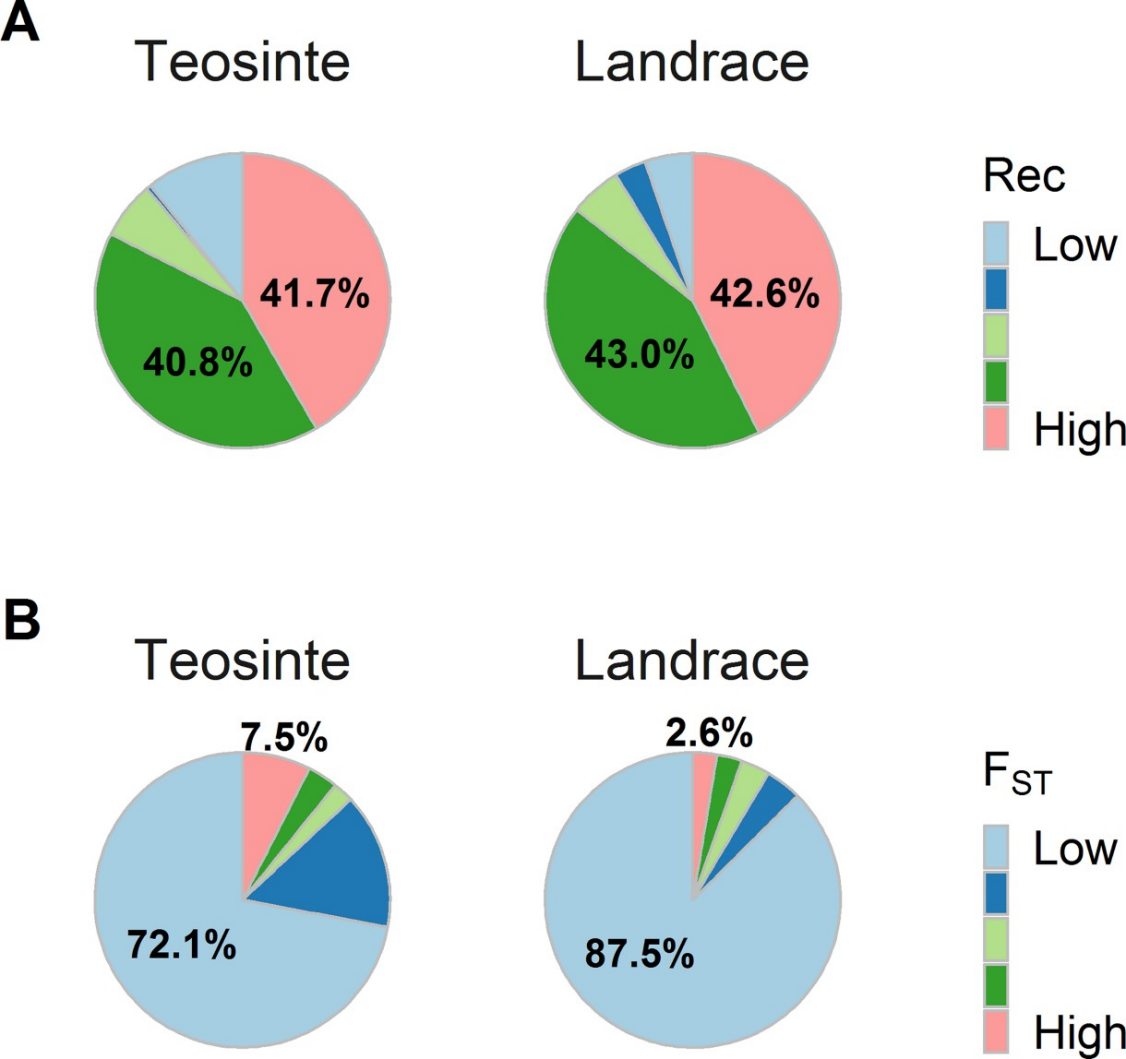
VCAP by different genomic features. (A) Five kinship matrices were calculated by SNPs in quintiles of the 10 kb recombination rates after accounting for gene density and then variances were estimated for each kinship. Rec stands for the residual of recombination rate after regression for gene density. (B) Five kinship matrices were calculated by SNPs in quintiles of the F_ST_ value and then variances were estimated for each kinship.

To assess how recombination would affect the heritable variance, we calculated the recombination rate in windows of 10 kb, defined quintiles of the 10 kb recombination rates after accounting for gene density, and used SNPs in each quintile to calculate kinship matrices in teosinte and maize landrace, respectively. By comparing the relative proportion of additive genetic variance across 18 traits, we observed that regions with the highest recombination rate (top quintile) explain a very large proportion of the additive genetic variance on average in both teosinte and maize landrace although they represent only 20% of the genome (Fig 7A), suggesting that high recombination regions harbored most of the variation upon which selection could act during domestication. While variance in these regions was somewhat depleted by domestication, it remains high in maize landrace.

#### Regions of the genome that are most divergent between teosinte and maize landrace explain more heritable variance in teosinte than in maize landrace

To assess the divergence between teosinte and maize landrace, we calculated F_ST_ [22] using 32.5 million SNPs that were either segregating in teosinte or maize landrace and obtained an average F_ST_ value in windows of 50 SNPs. The 50-SNP F_ST_ value ranges from 0 to 0.77 and has an average of 0.07. For VCA, we defined quintiles of F_ST_ and used SNPs in each quintile to calculate kinship matrices in teosinte and maize landrace, respectively. By comparing the relative proportion of additive genetic variance across 18 traits, we observed that the most divergent bin explains ~8% of the additive genetic variance on average in teosinte, which is more than ~3% in maize landrace (Fig 7B), suggesting that divergence is associated with depletion of additive genetic variants in the maize landrace.

### The inheritance of other traits in teosinte

Beyond the QTL analysis for 18 common core domestication traits in teosinte and maize landrace, we also identified QTL for seven additional traits that were scored in teosinte but not in maize landrace. These traits include BRAN, CULM, FCLN, FCLW, FCTR, SDDM and STAM (S3 Table). We detected a total of 222 QTL, ranging from 6 to 56 per trait (S1 Table). For these traits, we also observed that large effect QTL have low MAF (S2 Fig). For example, we detected a total of 19 QTL for CULM, which is a domestication trait related to vegetative gigantism. There were two large effect QTL for CULM which have a standardized additive effect greater than 1 phenotypic standard deviation (S1 Table). The largest effect QTL was detected on chromosome 1 (G/A, S1_178995091, P = 1.14E-27) but there are no obvious candidate genes nearby. Interestingly, we detected a QTL on chromosome 1 (G/A, S1_75448410, P = 3.06E-09) which is located within the inversion on chromosome 1 (*Inv1n*, B73 RefGen v4: 65.9–116.7 Mb) where a CULM QTL had previously been mapped [23].

A major change during maize domestication is the loss of seed dormancy from teosinte to maize. We detected six QTL for seed dormancy (SDDM). Among the QTL, there were three large effect QTL which have a standardized additive effect greater than 1 phenotypic standard deviation (S1 Table). The largest effect QTL was detected on chromosome 6 (G/T, S6_151838921, P = 1.10E-22) but there are no obvious candidate genes nearby. Interestingly, none of the six QTL exceed a MAF of 0.03, suggesting mutation-selection balance may play an important role in modulating genetic variation for SDDM. The common allele at five of the six QTL is associated with rapid germination.

## Discussion

Our results showed that the maize landrace population has far fewer QTL for domestication traits as compared to our teosinte population. Previous reports suggest that most domesticated crops have experienced a “domestication bottleneck” which reduced their nucleotide diversity relative to their wild ancestors [24]. For maize, there has been on average a reduction in nucleotide diversity of 30% as compared to teosinte and this reduction becomes much more severe (>65%) for genes under selection [25, 26]. Given that the domestication bottleneck and/or selection would cause the loss or fixation of many functional alleles at QTL, it was expected to find a reduced number of QTL in our maize landrace relative to teosinte.

Our observation of more and therefore novel QTL in teosinte compared to maize implies that teosinte should possess many useful alleles absent in maize. This represents an opportunity for breeding, as crop wild relatives have been extremely valuable resource to identify allelic variation by genome-wide association studies (GWAS) or quantitative trait locus (QTL) mapping approaches, and these alleles, if favorable, can be introduced into a domesticated background by marker-assisted selection [27, 28]. For maize-teosinte, a BC_2_S_3_ RIL (recombinant inbred line) population [29] and TeoNAM (teosinte nested association mapping) population [17] have been developed to comprehensively map QTL from teosinte. Using this BC_2_S_3_ population, a recent study demonstrated that fine-mapping QTL for maize leaf angle and then transferring the favorable teosinte allele into modern hybrids can enhance high-density maize grain yield [30]. Therefore, the favorable alleles of QTL that we detected in our teosinte population could be introduced into maize cultivars in the future.

Our observation that large effect alleles in both teosinte and maize landrace are rare fits expectations under mutation-selection balance [11]. Rare alleles of large effect would be rapidly removed if detrimental or driven to fixation if beneficial due to strong selection, and thus not be observed at middling frequencies. In contrast, alleles of very small effect experience weaker selection such that they move more slowly to fixation and can exhibit middling frequencies [31, 32]. Height in humans is an example from the literature of a trait apparently under mutation-selection balance, showing an inverse relationship between effect size and allele frequency [33]. Such an inverse relationship is also observed from a QTL study of inflorescence traits in maize, in which the number of large-effect QTL is very few and caused by low-frequency SNPs [34]. In our study, one example of a rare allele of large effect is the QTL for GW in teosinte (T/A, S4_184126058, P = 6.38E-50), where the MAF is 0.012 among the progeny and the minor allele comes from a single one of our 49 teosinte parents (PC_N57_ID2) (Fig 3A). Overall, the negative correlation between effect size and MAF in our data suggest an important role for mutation-selection balance in teosinte and maize.

While mutation-selection may be the principal force operating on QTL, we observed some hints that balancing selection may be present as well. In contrast to the expectation that large effect alleles are rare under mutation-selection balance, allele frequencies are expected to be more intermediate if balancing selection is operating [11]. An example of possible balancing selection in our data is the largest effect QTL for DTA in teosinte on chromosome 3 near *ZmMADS69* with an MAF of 0.48 (Fig 4A). Flowering time is a trait for which selection pressure may vary from year to year due to annual variation in rainfall and temperature [35]. In this situation, allelic fitness will oscillate from year to year, preventing segregating functional alleles at the locus from either being lost or fixed. However, we note that stabilizing selection on a phenotype which has recently shifted its optimum can also explain high frequency large effect alleles without invoking balancing selection [36].

For seed dormancy in teosinte, we expected to observe evidence for balancing selection in the form of functional alleles maintained at a high frequency since adaptation should favor a mix of phenotypes with seeds that germinate over a broad temporal range. Seed dormancy desynchronizes germination such that not all seed germinates the following season when environmental conditions for plant growth may be unsuitable in some years. Thus, selection should favor allelic diversity in wild species such that at least some seeds will be programed for delayed germination until future growth seasons [37]. Contrary to this expectation, we observed all six QTL detected for seed dormancy do not exceed a MAF of 0.03 which better fits a mutation-selection balance mechanism. In domesticated cultivars, it is desirable to have uniform germination to ensure synchronous maturity and therefore efficient harvesting by farmers [38, 39]. Accordingly, there is no seed dormancy in maize, and seeds, once planted, germinate readily.

Although large-effect QTL are rare, we observed the largest allele effects in maize (Fig 2). This reflects the differences in demography and selective forces acting on maize compared to teosinte. Teosinte traits are under selection because of their genetic correlations with fitness. Human selection in maize includes some similar fitness components (such as viability and fecundity), but the genetic correlations among maize traits are generally lower than among teosinte traits [8], and humans also select strongly for ear and kernel type, for example, possibly at the expense of natural fitness traits [40, 41]. Therefore, selection coefficients on fitness-related traits in maize are likely to be lower than in teosinte, reducing the effectiveness of selection on fixing or purging large-effect alleles. In combination with reduced selection, maize populations are managed by humans, who tend to plant seed from relatively few female parents [42], leading to greater genetic drift; but also migration is likely much more important in maize landraces than in teosinte because of active seed exchange among Mexican farmers [43, 44]. Recent migration could introduce novel alleles that were already fixed in other maize landraces, resulting in their segregation in the study population.

Another possibility is that the effective population size of our maize landrace is smaller than that of our teosinte population, so that selection would be more effective in teosinte. According to the Watterson estimator *θ* = 4*N_e_μ*, where N_e_ is the effective population size and μ is the per-generation mutation rate of the population [45], we calculated θ_T_ for the teosinte population and θ_M_ for the maize population using the common set of 4.2 million SNPs. The results show the mean Watterson’s θ in maize is 89.8% smaller than in teosinte (θ_M_ = 3.89E-04; θ_T_ = 4.33E-04). If we use the estimates of mutation rate in maize, which is around 3×10^−8^ per site per generation [46, 47], then N_e_ in our maize landrace is smaller than N_e_ in our teosinte population, thus selection would be less effective in maize landrace and cannot bring large effect alleles into loss or fixation.

We observed more dominance in maize landrace than teosinte as shown by an increase in D/A ratio (Fig 5), suggesting that the additive genetic variants were depleted at a faster rate than the dominance genetic variants during domestication. This is consistent with the observation of Yang et al. [8] that selection during domestication depleted the additive variance relative to the dominance variance. Population genetic theory has shown that traits that were subject to directional selection have significantly higher dominance estimates than traits that are not subject to strong selection [48, 49]. This theory aligns with our observation that maize landrace, which has been subject to strong selection, has an excess of dominant variants relative to teosinte for domestication traits.

Genomic scans can help to identify candidate genes for follow-up analyses. Our stepwise regression approach detected several strong candidate genes in both teosinte and maize landrace. (1) We detected a major effect QTL on chromosome 3 for flowering time in teosinte near *ZmMADS69*, which is under balancing selection. Several recent studies using maize-teosinte mapping populations have mapped a flowering time QTL containing this gene [17, 50, 51]. Another recent study further fine-mapped *ZmMADS69*, which functions as a flowering activator through the *ZmRap2*.*7*-*ZCN8* regulatory network and contributes to both long-day and short-day adaptation [52]. Notably, these studies investigated *ZmMADS69* in maize-teosinte crosses and they did not identify the causative site affecting flowering time. Therefore, our results could be useful for future investigation to discover novel alleles in teosinte alone, and determine the functional variant of *ZmMADS69*. (2) We also detected a major effect QTL on chromosome 4 for seed weight in teosinte. For this QTL, a candidate gene *TPP4* is near the QTL peak. *TPP4* is a fully redundant paralogue of *RAMOSA3* (*RA3*) and loss of both *RA3* and *TPP4* in maize leads to reduced meristem determinacy and more inflorescence branching [53]. (3) Finally, we detected two rough sheath genes as candidate QTL of plant height: *rough sheath1* in landrace and *rough sheath2* in teosinte. Both genes have been reported to affect plant architecture including plant height [20, 21]. It may be worth exploring natural variation in these genes and how domestication has acted on them.

We observed that a large proportion (~75%) of the additive heritability is missing in the sense that it cannot be ascribed to the detected QTL. This is true for both our teosinte and maize landrace populations across ten domestication traits (Fig 6). Missing heritability has been widely investigated in human studies and a well-characterized example is height [54]. The heritability of height has been estimated to be ~0.8 by twin studies, while the ~50 detected variants (QTL) first associated with height explained only ~5% of the phenotypic variance [55]. Subsequent larger studies identified 180 height genes that accounted for only 10% of the phenotypic variation [56] and 697 genes that explained only 20% of the heritable variation [57]. There are three main hypotheses regarding missing heritability [58, 59]: (1) missing heritability is largely due to rare variants of large effect, (2) the majority of missing heritability is attributable to common variants of very small effect, and (3) heritability estimates from classic twin studies are biased upward so there actually is no missing heritability.

For our study, large effect but rare variants should be detected because only 49 or 40 parents contributed to the 4,455 or 4,398 progeny. By our design, variants of large effect that are rare in nature become relatively common in our mapping population. Despite this design, we detected relatively few rare QTL of large effect and all QTL together explain only about 25% of the additive variance. Thus, we can exclude the hypothesis that rare alleles of large effect explain an appreciable fraction of the missing heritability. Similarly, we used a robust design and common-garden protocol to estimate trait heritabilities [8], thus it seems unlikely that our heritabilities are biased upward. Moreover, our heritability estimates and QTL detection were performed on precisely the same populations. Thus, our data argue strongly that most heritable variation in both teosinte and landrace is controlled by a mix of rare and common alleles of very small effect sizes below the threshold for detection as individual QTL–that is the infinitesimal model [60, 61].

Our variance component analysis shows that SNPs in regions of high recombination explain most heritable variance in both teosinte and maize landrace, suggesting recombination facilitates the maintenance of additive variation in our populations. It has been proposed that there is an interaction between natural selection and the recombination rate, known as the Hill-Robertson effect, such that there is a reduction in the effectiveness of natural selection in regions of low recombination [62, 63]. With low recombination, selection on one locus will drag variants at linked loci to fixation thereby reducing variation in the region [64]. A well-characterized example is reduced codon bias in regions of low recombination in Drosophila, which supports that natural selection is less effective against slightly disadvantageous mutations in regions lacking recombination [65]. Our observation of greater additive variance being associated with SNPs in regions of high recombination can be explained by the Hill-Robertson effect although this effect may be confounded with the Bulmer effect [11], as we observed the lower recombination regions have a bit more LD (S4 Table).

We also show that SNPs in regions of high divergence (high F_ST_) between maize and teosinte explain more variance in teosinte than maize landrace, suggesting variants in these regions were either removed or brought to fixation during domestication, congruent with the results of Xue et al. [66]. A common feature of crop genomes is regions of reduced nucleotide diversity in the crop relative to its wild progenitor [5, 25, 28]. One example is a selective sweep of ~60–90 kb 5’ to the maize domestication gene *tb1* that has an extended region of low nucleotide diversity [67, 68]. Another example is a large region on chromosome 10 in maize that was the target of strong selection during domestication, producing a 1.1 Mb segment with >15 genes that lost essentially all genetic diversity [69]. Such regions of high diversity in teosinte and low diversity in maize result in higher nucleotide divergence between maize and teosinte (S3 Fig). Thus, the observation that SNPs in regions of high divergence explain more genetic variance in teosinte accords with the expectation that many variants should have been either removed or fixed during and after domestication bottleneck.

The genetic dissection of crop domestication has been a focus of agronomists and evolutionary biologists ever since the rediscovery of Mendel’s law of inheritance. In this enterprise, diverse approaches have been utilized including classic Mendelian genetics, phylogenetics, population genetics, genomic scans for selection, analysis of differential gene expression, QTL mapping, association mapping, and cloning/charactering major genes controlling domestication traits [4, 5, 7]. In this paper, we take a novel approach of comparing the genetics architecture of 18 domestication traits in maize and its ancestor teosinte using matched populations. From our analyses, we infer that there has been a substantial depletion of quantitative genetic variants in maize relative to teosinte in terms of the numbers of segregating QTL for domestication traits. At the same time, the degree of dominance of segregating QTL has increased in maize, likely a consequence of selective removal of additive variants. We observed that regions of the genome that are strongly differentiated between teosinte and maize (high F_ST_) explain less quantitative variation in maize than teosinte, suggesting that, in these regions, allelic variants were brought to (or near) fixation during domestication. Finally, we observed that about 75% of the additive variance in both teosinte and maize is missing in the sense that it cannot be ascribed to detectable QTL and only 25% of variance maps to specific QTL. This latter result suggests that morphological evolution during domestication is largely attributable to very large numbers of QTL of very small effect.

## Materials and methods

### Population, genotype and phenotype

Data for our analyses were previously published, and so details concerning population construction, plant growth, phenotyping, and SNP genotyping by Genotype-by-Sequence (GBS) technology for both our teosinte and landrace populations can be found in Yang et al. [8]. Briefly, a population of 70 teosinte plants from the near the town of Palmar Chico in Balsas river drainage of Mexico and a population of 55 maize landrace (Tuxpeño) plants from a nearby location were sampled. DNA from all 125 plants was used for whole-genome-sequencing (WGS). Of the 70 teosinte plants, 49 were used as parents and selfed and intermated to produce a total of 4,455 teosinte progeny. Similarly, of the 55 landrace plants, 40 were used as parents and selfed and intermated to produce a total of 4,398 maize landrace progeny. The parentage of progeny was determined using the GBS data of the parents and progeny.

The teosinte and landrace progeny were grown in neighboring fields near Homestead, Florida over during two winter seasons (2013–14 and 2014–15). Eighteen domestication traits were scored on both the teosinte and landrace progeny and these were the focus of the work of Yang et al. [8] (Table 1). Seven additional traits were scored in teosinte alone and analyzed in this paper (S3 Table). For GBS, a total of 34,899 SNPs was scored for teosinte and 40,255 SNPs for maize landrace. Yang et al. [8] estimated a variety of quantitative genetic parameters for these populations including: additive genetic variance, dominance genetic variance, phenotypic variance, genetic-by-environmental variance, selection intensity, genetic correlation matrix, and genetic variance-covariance matrix. All phenotype and genotype data from Yang et al. [8] are available at www.pnas.org/lookup/suppl/doi:10.1073/pnas.1820997116/-/DCSupplemental and https://doi.org/10.6084/m9.figshare.7655588.

In this paper, we added to this dataset by determining the WGS for all 125 teosinte and landrace parent plants. We extracted a total of 18 million and 21 million SNPs from the WGS data for teosinte and maize landrace, respectively, after removing sites with missing rate above 10% and heterozygosity rate above 60% in the parents. Using skim-WGS of selected progeny of both teosinte and landrace, we phased the SNPs in the 49 teosinte parents and 40 landrace parents that contributed to the 4455 teosinte and 4398 landrace progenies. Yang et al. [8] reported the recombination breakpoints on all chromosomes for all progeny as defined by the GBS SNPs. Using these breakpoint locations and phased WGS SNPs of the parents, we were able to project the WGS SNPs of the parents onto all progeny. This process resulted in a total of 32.5 million SNPs with 17.8 million segregating SNPs in teosinte and 18.9 million segregating SNPs in maize landrace, of which 4.2 million are shared in both populations. These SNPs are available via Cyverse Data Commons: https://doi.org/10.25739/sa4n-b482. Details on how this was accomplished are presented in S1 Appendix.

### Genome-Wide Association Study (GWAS)

GWAS was performed by a scan of the genome using stepwise regression analysis. We first fit a general linear model (GLM) to reduce SNP number. The model included field variables, the inbreeding coefficient, and the first 50 principal components (PCs) based on the GBS SNPs as covariates. We then selected the SNP with the lowest P value within each 200-SNP bin along each chromosome, and used the reduced set of SNPs for stepwise regression to map quantitative trait loci (QTLs) for each trait by fitting an additive plus dominance model with the same covariates as used for GLM for stepwise regression. Both GLM and stepwise regression were implemented with TASSEL5 software [70]. Details on the scanning model are presented in S1 Appendix.

### Variance Component Analysis (VCA)

To estimate the proportion of trait additive genetic variation associated with different classes of SNPs, we used a procedure to estimate variance components associated with different subsets of the SNPs [66, 71–73]. In brief, variance component analysis (VCA) was done by (1) classifying SNPs into subsets based on a hypothesis of interest, (2) generating kinship matrices for each subset using TASSEL5 [70], and (3) fitting these kinship matrices along with phenotypic data into LDAK5 (http://dougspeed.com/ldak/), using a generalized restricted maximum likelihood (REML) solver to partition the genetic variance (heritability, h^2^) into the proportion accounted for by each SNP subset [72, 73]. Details on how we performed VCA in this study are presented in S1 Appendix.

## Supporting information

S1 Appendix Supporting materials and methods. (PDF)

S1 Fig The relationship between effect size and MAF for all SNPs in 18 traits. (PDF)

S2 Fig The relationship between effect size and MAF for 222 QTL in seven teosinte-only traits. (PDF)

S3 Fig The relationship between nucleotide diversity and F_ST_. (PDF)

S1 Table QTLs detected in this study. (XLSX)

S2 Table Summary of heritability. (PDF)

S3 Table Descriptions for seven teosinte-only traits analyzed in this study. (PDF)

S4 Table Comparison of LD for regions of different recombination rate. (PDF)

S5 Table Number of progeny used for phasing teosinte parents. (PDF)

S6 Table Number of progeny used for phasing maize landrace parents. (PDF)

S1 Text Perl script for phasing parents. (PDF)

## References

[1] Darwin C. On the Origin of Species by Means of Natural Selection, or the Preservation of Favoured Races in the Struggle for Life. H. Milford; Oxford University Press; 1859.

[2] Burger JC, Chapman MA, Burke JM. Molecular insights into the evolution of crop plants. Am J Bot. 2008; 95: 113–122. 10.3732/ajb.95.2.113 21632337

[3] Doebley JF, Gaut BS, Smith BD. The molecular genetics of crop domestication. Cell. 2006; 127: 1309–1321. 10.1016/j.cell.2006.12.006 17190597

[4] Gross BL, Olsen KM. Genetic perspectives on crop domestication. Trends Plant Sci. 2010; 15: 529–537. 10.1016/j.tplants.2010.05.008 20541451 PMC2939243

[5] Meyer RS, Purugganan MD. Evolution of crop species: genetics of domestication and diversification. Nat Rev Genet. 2013; 14: 840–852. 10.1038/nrg3605 24240513

[6] Olsen KM, Wendel JF. A bountiful harvest: genomic insights into crop domestication phenotypes. Ann Rev Plant Biol. 2013; 64: 47–70. 23451788 10.1146/annurev-arplant-050312-120048

[7] Swinnen G, Goossens A, Pauwels L. Lessons from domestication: targeting cis-regulatory elements for crop improvement. Trends Plant Sci. 2016; 21: 506–515. 10.1016/j.tplants.2016.01.014 26876195

[8] Yang CJ, Samayoa LF, Bradbury PJ, Olukolu BA, Xue W, York AM, et al The genetic architecture of teosinte catalyzed and constrained maize domestication. Proc Natl Acad Sci USA. 2019; 116: 5643–5652. 10.1073/pnas.1820997116 30842282 PMC6431195

[9] Matsuoka Y, Vigouroux Y, Goodman MM, Sanchez J, Buckler E, Doebley J. A single domestication for maize shown by multilocus microsatellite genotyping. Proc Natl Acad Sci USA. 2002; 99: 6080–6084. 10.1073/pnas.052125199 11983901 PMC122905

[10] Piperno DR, Ranere AJ, Holst I, Iriarte J, Dickau R. Starch grain and phytolith evidence for early ninth millennium B.P. maize from the Central Balsas River Valley, Mexico. Proc Natl Acad Sci USA. 2009; 106: 5019–5024. 10.1073/pnas.0812525106 19307570 PMC2664021

[11] Walsh B, Lynch M. Evolution and Selection of Quantitative Traits. Oxford University Press; 2018.

[12] Jiao Y, Peluso P, Shi J, Liang T, Stitzer MC, Wang B, et al Improved maize reference genome with single-molecule technologies. Nature. 2017; 546: 524 10.1038/nature22971 28605751 PMC7052699

[13] Ou S, Liu J, Chougule KM, Fungtammasan A, Seetharam A, Stein J, et al Effect of sequence depth and length in long-read assembly of the maize inbred NC358. BioRxiv [Preprint]. 2019 bioRxiv 858365 [posted 2019 Nov 29; revised 2019 Dec 3; cited 2020 Feb 10]: [24 p.]. Available from: https://www.biorxiv.org/content/10.1101/858365v2 10.1101/858365 PMC7211024 32385271

[14] Springer NM, Anderson SN, Andorf CM, Ahern KR, Bai F, Barad O, et al The maize W22 genome provides a foundation for functional genomics and transposon biology. Nat Genet. 2018; 50: 1282 10.1038/s41588-018-0158-0 30061736

[15] Sun S, Zhou Y, Chen J, Shi J, Zhao HM, Zhao HN, et al Extensive intraspecific gene order and gene structural variations between Mo17 and other maize genomes. Nat Genet. 2018; 50: 1289 10.1038/s41588-018-0182-0 30061735

[16] Bukowski R, Guo X, Lu Y, Zou C, He B, Rong Z, et al Construction of the third-generation *Zea mays* haplotype map. Gigascience. 2018; 7: gix134. 10.1093/gigascience/gix134 PMC5890452 29300887

[17] Chen Q, Yang CJ, York AM, Xue W, Daskalska LL, DeValk CA, et al TeoNAM: a nested association mapping population for domestication and agronomic trait analysis in maize. Genetics. 2019; 213: 1065–1078. 10.1534/genetics.119.302594 31481533 PMC6827374

[18] Yu J, Holland JB, McMullen MD, Buckler ES. Genetic design and statistical power of nested association mapping in maize. Genetics. 2008; 178: 539–551. 10.1534/genetics.107.074245 18202393 PMC2206100

[19] Pyhäjärvi T, Hufford MB, Mezmouk S, Ross-Ibarra J. Complex patterns of local adaptation in teosinte. Genome Biol Evol. 2013; 5: 1594–1609. 10.1093/gbe/evt109 23902747 PMC3787665

[20] Schneeberger R, Tsiantis M, Freeling M, Langdale JA. The *rough sheath2* gene negatively regulates homeobox gene expression during maize leaf development. Development. 1998; 125: 2857–2865. 9655808 10.1242/dev.125.15.2857

[21] Becraft PW, Freeling M. Genetic analysis of *Rough sheath1* developmental mutants of maize. Genetics. 1994; 136: 295–311. 8138166 10.1093/genetics/136.1.295 PMC1205781

[22] Weir BS, Cockerham CC. Estimating F‐statistics for the analysis of population structure. Evolution. 1984; 38: 1358–1370. 10.1111/j.1558-5646.1984.tb05657.x 28563791

[23] Fang Z, Pyhäjärvi T, Weber AL, Dawe RK, Glaubitz JC, González JD, et al Megabase-scale inversion polymorphism in the wild ancestor of maize. Genetics. 2012; 191: 883–894. 10.1534/genetics.112.138578 22542971 PMC3389981

[24] Buckler ES, Thornsberry JM, Kresovich S. Molecular diversity, structure and domestication of grasses. Genet Res. 2001; 77: 213–218. 11486504 10.1017/s0016672301005158

[25] Hufford MB, Xu X, Van Heerwaarden J, Pyhäjärvi T, Chia JM, Cartwright RA, et al Comparative population genomics of maize domestication and improvement. Nat Genet. 2012; 44: 808 10.1038/ng.2309 22660546 PMC5531767

[26] Wright SI, Bi IV, Schroeder SG, Yamasaki M, Doebley JF, McMullen MD, et al The effects of artificial selection on the maize genome. Science. 2005; 308: 1310–1314. 10.1126/science.1107891 15919994

[27] Dempewolf H, Baute G, Anderson J, Kilian B, Smith C, Guarino L. Past and future use of wild relatives in crop breeding. Crop Sci. 2017; 57: 1070–1082.

[28] Tanksley SD, McCouch SR. Seed banks and molecular maps: unlocking genetic potential from the wild. Science. 1997; 277: 1063–1066. 10.1126/science.277.5329.1063 9262467

[29] Shannon LM, Chen Q, Doebley JF. A BC2S3 maize-teosinte RIL population for QTL mapping. Maize Genet Coop News Lett. 2019; 93.

[30] Tian J, Wang C, Xia J, Wu L, Xu G, Wu W, et al Teosinte ligule allele narrows plant architecture and enhances high-density maize yields. Science. 2019; 365: 658–664. 10.1126/science.aax5482 31416957

[31] Desai MM, Fisher DS. Beneficial mutation–selection balance and the effect of linkage on positive selection. Genetics. 2007; 176: 1759–1798. 10.1534/genetics.106.067678 17483432 PMC1931526

[32] Perfeito L, Fernandes L, Mota C, Gordo I. Adaptive mutations in bacteria: high rate and small effects. Science. 2007; 317: 813–815. 10.1126/science.1142284 17690297

[33] Kemper KE, Visscher PM, Goddard ME. Genetic architecture of body size in mammals. Genome Biol. 2012; 13: 244 10.1186/gb-2012-13-4-244 22546202 PMC3446298

[34] Brown PJ, Upadyayula N, Mahone GS, Tian F, Bradbury PJ, Myles S, et al Distinct genetic architectures for male and female inflorescence traits of maize. PLoS Genet. 2011; 7: e1002383 10.1371/journal.pgen.1002383 22125498 PMC3219606

[35] Troth A, Puzey JR, Kim RS, Willis JH, Kelly JK. Selective trade-offs maintain alleles underpinning complex trait variation in plants. Science. 2018; 361: 475–478. 10.1126/science.aat5760 30072534

[36] Stetter MG, Thornton K, Ross-Ibarra J. Genetic architecture and selective sweeps after polygenic adaptation to distant trait optima. PLoS Genet. 2018; 14: e1007794 10.1371/journal.pgen.1007794 30452452 PMC6277123

[37] Avendaño López AN, de Jesús Sánchez González J, Ruíz Corral JA, De La Cruz Larios L, Santacruz-Ruvalcaba F, Sánchez Hernández CV, et al Seed dormancy in Mexican teosinte. Crop Sci. 2011; 51: 2056–2066.

[38] Née G, Xiang Y, Soppe WJ. The release of dormancy, a wake-up call for seeds to germinate. Curr Opin Plant Biol. 2017; 35: 8–14. 10.1016/j.pbi.2016.09.002 27710774

[39] Rendón-Anaya M, Herrera-Estrella A. The advantage of parallel selection of domestication genes to accelerate crop improvement. Genome Biol. 2018; 19: 147 10.1186/s13059-018-1537-7 30266085 PMC6161459

[40] Hernández Xolocotzi E. Maize and man in the greater southwest. Econ Bot. 1985; 39: 416–430.

[41] Logan AL, Hastorf CA, Pearsall DM. “Let’s drink together”: early ceremonial use of maize in the Titicaca Basin. Lat Am Antiq. 2012; 23: 235–258.

[42] Louette D, Charrier A, Berthaud J. In situ conservation of maize in Mexico: genetic diversity and maize seed management in a traditional community. Econ Bot. 1997; 51: 20–38.

[43] Perales H, Brush SB, Qualset CO. Dynamic management of maize landraces in Central Mexico. Econ Bot. 2003; 57: 21.

[44] Pressoir G, Berthaud J. Patterns of population structure in maize landraces from the Central Valleys of Oaxaca in Mexico. Heredity. 2004; 92: 88 10.1038/sj.hdy.6800387 14666127

[45] Watterson GA. On the number of segregating sites in genetical models without recombination. Theor Popul Biol. 1975; 7:256–276. 10.1016/0040-5809(75)90020-9 1145509

[46] Clark RM, Tavaré S, Doebley J. Estimating a nucleotide substitution rate for maize from polymorphism at a major domestication locus. Mol Biol Evol. 2005; 22: 2304–2312. 10.1093/molbev/msi228 16079248

[47] Yang N, Xu XW, Wang RR, Peng WL, Cai L, Song JM, et al Contributions of Zea mays subspecies mexicana haplotypes to modern maize. Nat Commun. 2017; 8: 1–10. 10.1038/s41467-016-0009-6 29187731 PMC5707364

[48] Crnokrak P, Roff DA. Dominance variance: associations with selection and fitness. Heredity. 1995; 75: 530.

[49] Merilä J, Sheldon BC. Genetic architecture of fitness and nonfitness traits: empirical patterns and development of ideas. Heredity. 1999; 83: 103–109. 10.1046/j.1365-2540.1999.00585.x 10469197

[50] Li D, Wang X, Zhang X, Chen Q, Xu G, Xu D, et al The genetic architecture of leaf number and its genetic relationship to flowering time in maize. New Phytol. 2016; 210: 256–268. 10.1111/nph.13765 26593156 PMC5063108

[51] Shannon LM. The genetic architecture of maize domestication and range expansion. [PhD Dissertation] The University of Wisconsin-Madison; 2012.

[52] Liang Y, Liu Q, Wang X, Huang C, Xu G, Hey S, et al *ZmMADS69* functions as a flowering activator through the *ZmRap2*.*7*-*ZCN8* regulatory module and contributes to maize flowering time adaptation. New Phytol. 2019; 221: 2335–2347. 10.1111/nph.15512 30288760

[53] Claeys H, Vi SL, Xu X, Satoh-Nagasawa N, Eveland AL, Goldshmidt A, et al Control of meristem determinacy by trehalose 6-phosphate phosphatases is uncoupled from enzymatic activity. Nat Plants. 2019; 5: 352 10.1038/s41477-019-0394-z 30936436 PMC7444751

[54] Young AI. Solving the missing heritability problem. PLoS Genet. 2019; 15: e1008222 10.1371/journal.pgen.1008222 31233496 PMC6611648

[55] Visscher PM. Sizing up human height variation. Nat Genet. 2008; 40: 489 10.1038/ng0508-489 18443579

[56] Allen HL, Estrada K, Lettre G, Berndt SI, Weedon MN, Rivadeneira F, et al Hundreds of variants clustered in genomic loci and biological pathways affect human height. Nature. 2010; 467: 832 10.1038/nature09410 20881960 PMC2955183

[57] Wood AR, Esko T, Yang J, Vedantam S, Pers TH, Gustafsson S, et al Defining the role of common variation in the genomic and biological architecture of adult human height. Nat Genet. 2014; 46: 1173 10.1038/ng.3097 25282103 PMC4250049

[58] Eichler EE, Flint J, Gibson G, Kong A, Leal SM, Moore JH, et al Missing heritability and strategies for finding the underlying causes of complex disease. Nat Rev Genet. 2010; 11: 446 10.1038/nrg2809 20479774 PMC2942068

[59] Yang J, Bakshi A, Zhu Z, Hemani G, Vinkhuyzen AA, Lee SH, et al Genetic variance estimation with imputed variants finds negligible missing heritability for human height and body mass index. Nat Genet. 2015; 47: 1114 10.1038/ng.3390 26323059 PMC4589513

[60] Fisher RA. The correlation between relatives on the supposition of mendelian inheritance. Trans R Soc Edinb. 1918; 52: 399–433.

[61] Wainschtein P, Jain DP, Yengo L, Zheng Z, Cupples LA, Shadyab AH, et al Recovery of trait heritability from whole genome sequence data. BioRxiv [Preprint]. 2019 bioRxiv 588020 [posted 2019 Mar 25; cited 2020 Feb 10]: [23 p.]. Available from: https://www.biorxiv.org/content/10.1101/588020v1 10.1101/588020

[62] Felsenstein J. The evolutionary advantage of recombination. Genetics. 1974; 78: 737–756. 4448362 10.1093/genetics/78.2.737 PMC1213231

[63] Hill WG, Robertson A. The effect of linkage on limits to artificial selection. Genet Res. 1966; 8: 269–294. 5980116

[64] Cutter AD, Payseur BA. Genomic signatures of selection at linked sites: unifying the disparity among species. Nat Rev Genet. 2013; 14: 262 10.1038/nrg3425 23478346 PMC4066956

[65] Kliman RM, Hey J. Reduced natural selection associated with low recombination in *Drosophila melanogaster*. Mol Biol Evol. 1993; 10: 1239–1258. 10.1093/oxfordjournals.molbev.a040074 8277853

[66] Xue S, Bradbury PJ, Casstevens T, Holland JB. Genetic architecture of domestication-related traits in maize. Genetics. 2016; 204: 99–113. 10.1534/genetics.116.191106 27412713 PMC5012408

[67] Clark RM, Linton E, Messing J, Doebley JF. Pattern of diversity in the genomic region near the maize domestication gene *tb1*. Proc Natl Acad Sci USA. 2004; 101: 700–707. 10.1073/pnas.2237049100 14701910 PMC321743

[68] Studer A, Zhao Q, Ross-Ibarra J, Doebley J. Identification of a functional transposon insertion in the maize domestication gene *tb1*. Nat Genet. 2011; 43: 1160 10.1038/ng.942 21946354 PMC3686474

[69] Tian F, Stevens NM, Buckler ES. Tracking footprints of maize domestication and evidence for a massive selective sweep on chromosome 10. Proc Natl Acad Sci USA. 2009; 106: 9979–9986. 10.1073/pnas.0901122106 19528660 PMC2702805

[70] Bradbury PJ, Zhang Z, Kroon DE, Casstevens TM, Ramdoss Y, Buckler ES. TASSEL: software for association mapping of complex traits in diverse samples. Bioinformatics. 2007; 23: 2633–2635. 10.1093/bioinformatics/btm308 17586829

[71] Rodgers-Melnick E, Vera DL, Bass HW, Buckler ES. Open chromatin reveals the functional maize genome. Proc Natl Acad Sci USA. 2016; 113: E3177–E3184. 10.1073/pnas.1525244113 27185945 PMC4896728

[72] Speed D, Balding DJ. MultiBLUP: improved SNP-based prediction for complex traits. Genome Res. 2014; 24: 1550–1557. 10.1101/gr.169375.113 24963154 PMC4158754

[73] Speed D, Hemani G, Johnson MR, Balding DJ. Improved heritability estimation from genome-wide SNPs. Am J Hum Genet. 2012; 91: 1011–1021. 10.1016/j.ajhg.2012.10.010 23217325 PMC3516604

