# Supplementary material for: The genetic architecture of the maize progenitor, teosinte, and how it was altered during maize domestication
Source: PLoS Genet. 2020 May 14;16(5):e1008791. doi: 10.1371/journal.pgen.1008791 (PMC7266358; doi:10.1371/journal.pgen.1008791)
Supplement: S1 Appendix — (PDF) [file pgen.1008791.s001.pdf]

PLOS | GENETICS

Supporting information for:

The genetic architecture of the maize progenitor, teosinte, and how it was altered during maize domestication

Qiuyue Chen, Luis Fernando Samayoa, Chin Jian Yang, Peter J. Bradbury, Bode A. Olukolu, Michael A. Neumeyer, Maria Cinta Romay, Qi Sun, Anne Lorant, Edward S. Buckler, Jeffrey Ross-Ibarra, James B. Holland, John F. Doebley*

* Corresponding author. E-mail: jdoebley@wisc.edu

DOI: 10.1371/journal.pgen.1008791

This pdf file includes:

Materials and Methods

References

Materials and Methods

The core data for our analyses were previously published, and so details concerning population construction, plant growth, phenotyping, and SNP genotyping by Genotype-by-Sequence (GBS) technology for both our teosinte and landrace populations can be found in Yang et al. [1]. Briefly, a population of 70 teosinte plants from the near the town of Palmar Chico in Balsas river drainage of Mexico and a population of 55 maize landrace (Tuxpeño) plants from a nearby location were sampled. DNA from all 125 plants was used for whole-genome-sequencing (WGS) (see below). Of the 70 teosinte plants, 49 were used as parents and selfed and intermated to produce a total of 4,455 teosinte progeny. Similarly, of the 55 landrace plants, 40 were used as parents and selfed and intermated to produce a total of 4,398 maize landrace progeny. The teosinte population has 49 selfed families with family size ranging from 3 to 95 progeny and 288 outcross families with family size ranging from 1 to 75 progeny. The maize landrace population has 34 selfed families with family size ranging from 1 to 125 progeny and 55 outcross families with family size ranging from 6 to 141 progeny. The parentage of progeny was determined using the GBS data of the parents and progeny.

The teosinte and landrace progeny were grown in neighboring fields near Homestead, Florida over during two winter seasons (2013-14 and 2014-15). Eighteen domestication traits were scored on both the teosinte and landrace progeny and these were the focus of the work of Yang et al. [1] (Table 1). Some additional traits were scored in teosinte alone, seven of which are analyzed in this paper (S3 Table). For GBS, a total of 34,899 SNPs was scored for teosinte and 40,255 SNPs for maize landrace. Yang et al. [1] estimated a variety of quantitative genetic parameters for these populations including: additive genetic variance, dominance genetic variance, phenotypic variance, genetic-by-environmental variance, selection intensity, genetic correlation matrix, and genetic variance-covariance matrix. All phenotype and genotype data from Yang et al. [1] are available at www.pnas.org/lookup/suppl/doi:10.1073/pnas.1820997116/-/DCSupplemental and https://doi.org/10.6084/m9.figshare.7655588.

In this paper, we added to this dataset by determining the WGS for all 125 teosinte and landrace parent plants. We extracted a total of 18 million and 21 million SNPs from

the WGS data for teosinte and maize landrace, respectively, after removing sites with missing rate above 10% and heterozygosity rate above 60% in the parents. Using skim-WGS of selected progeny of both teosinte and landrace, we phased the SNPs in the 49 teosinte parents and 40 landrace parents that contributed to the 4455 teosinte and 4398 landrace progenies. Yang et al. [1] reported the recombination breakpoints on all chromosomes for all progeny as defined by the GBS SNPs. Using these breakpoint locations and phased WGS SNPs of the parents, we were able to project the WGS SNPs of the parents onto all progeny. This process resulted in a total of 32.5 million SNPs with 17.8 million segregating SNPs in teosinte and 18.9 million segregating SNPs in maize landrace, of which 4.2 million are shared in both populations. Details on how this was accomplished are presented below.

DNA Extraction, Library Construction, WGS

Leaf tissue samples from the parents and progeny of teosinte and maize landrace were collected for DNA isolation using several different methods. For the teosinte parents, 100 – 350 mg leaf tissue samples were collected depending on the DNA isolation protocol used, which was either DNeasy® Plant Kit (Qiagen Inc., Germantown, MD) or modified CTAB protocol [2]. Due to frequent poor yield with the DNAeasy® kit, DNAs were pooled from three to five leaf tissue samples for each low yield parent. DNAs isolated from the modified CTAB protocol were sufficient so no pooling was required. For the maize landrace parents, 100 mg leaf tissue samples were collected and lyophilized prior to DNA isolation with the same kit but without pooling. All of the tissue samples were lyophilized prior to DNA isolation using DNeasy® Plant Kit (Qiagen Inc., Germantown, MD).

For WGS of parents, 1ug of DNA was fragmented using a bioruptor (Diagenode) with cycles of 30 seconds on, 30 seconds off. DNA fragments were then prepared for Illumina sequencing. First, DNA fragments were repaired with the End-Repair enzyme mix (New England Biolabs). A deoxyadenine triphosphate was added at each 3’ end with the Klenow fragment (New England Biolabs). Illumina TruSeq adapters (Affymetrix) were then added with the Quick ligase kit (New England Biolabs). Between each enzymatic step, DNA was washed with Sera-Mags SpeedBeads (Fisher Scientific). The

70 teosinte parents and 38 teosinte progeny were sequenced at UC Berkley on the HiSeq2500 for paired-end 100 base reads (PE100). The 55 maize landraces were sequenced at the UC Davis Genome Center on the HiSeq3000 for PE150. For parents, all the samples were sequenced to achieve ~20x coverage. For the 38 teosinte progeny, the coverage is ~1x.

For skim sequencing of other progeny, DNA from the selected progeny was used to construct high throughput Illumina Nextera libraries and sequenced at 24 plex in one lane of Hiseq X Ten each, providing approximately 2x coverage for each sample (https://www.illumina.com/). Raw data is available at the NCBI BioProject accession: PRJNA616247.

WGS Read Alignment and Variant Calling

Illumina sequencing reads were aligned to maize reference genome B73 AGPv4 [3] using BWA-MEM [4] (version 0.7.13) with default setting, then sorted and indexed with Picard tools (http://broadinstitute.github.io/picard, version 2.8.2). Sentieon [5] (version 201704.03) was used for removing PCR duplicates, and for variant calling. Sentieon Haplotyper algorithm was used as described in the Sentieon manual. Only reads with mapping quality 60 or higher were used, with call_conf set to 10 and emit_conf set to 10.

Parent Phasing and SNP Projection to Progeny

The WGS data for teosinte and maize landrace parents were phased separately. First, the data were filtered to remove non-biallelic sites and insertion-deletion (indel) polymorphisms. We also filtered out SNPs with very high depth in the parents (≥95% quantile across all SNPs), suggesting that these SNPs represent duplicated regions of the genome. After filtering, we phased the parents at heterozygous sites using the skim-WGS data from selected progeny. For the selfed progeny, the homozygous regions of the genome are known from the GBS data and only SNPs in these homozygous regions were used to phase the parents. For each parent with enough selfed progeny, four selfed progeny were selected to provide maximum coverage of the parent by the homozygous regions of the progeny. In most cases, four progeny could be chosen to cover more than 99% of the genome. Phasing using selfed progeny used

the fact that each homozygous segment came from a single parental chromosome and the other chromosome could be determined by subtraction. For outcross progeny, only sites that were heterozygous in one parent and homozygous in the other can be used to phase chromosomes in the heterozygous parent. When the progeny is determined to carry the minor allele from the heterozygote, then it can be used to tag one of the parental chromosomes. In addition, with skim sequence which have read depth of 1 for many sites, only when the minor allele is observed can that site be called. On average that is about half of the potential sites. As a result, more outcross progeny are needed to provide coverage similar to selfed progeny. We picked outcrosses to a parent with enough selfs so if that parent with selfs can be phased well, then maybe we can phase the parent with outcrosses to it decently.

In summary, we sequenced 230 progeny for teosinte including 195 selfs and 35 outcrosses (S5 Table) and 192 progeny for maize landrace including 139 selfs and 53 outcrosses (S6 Table). We phased teosinte parents using only selfed progeny as each parent had a sufficient number of selfed progeny, i.e., all parents had more than four selfs except for one. For maize landrace parents, we used both selfed and outcrossed progeny to phase the parents because there were too few or no selfed progeny for some parents, i.e., only 32 parents had enough selfs. The WGS SNPs of each parent were phased using data from single progeny at a time, after which we compared the phasing of the parent from different progeny and set those sites that were inconsistently phased as missing. The whole phasing process was done using an in-house Perl script (S1 Text). After phasing, we filtered the WGS SNPs to remove sites with missing rate above 10% and heterozygosity rate above 60% in the parents.

With the known parentage of progeny and recombination breakpoint locations, the phased WGS SNPs of the parents were projected onto progeny with the *projection* function implemented in TASSEL5 [6]. After projection, we filtered the WGS data for teosinte and maize landrace separately. We removed the following: (1) sites with minor allele frequency (maf) among the progeny below 0.001, (2) sites with missing rate above 20%, and (3) sites with no polymorphism. The final result was the set of 17.8

million SNPs for the 4455 teosinte progeny and 18.9 million SNPs for the 4398 landrace progeny that were used for our quantitative genetic analyses.

Phenotyping

Yang et al. [1] previously described how the 18 core domestication traits were phenotyped. Seven additional traits scored in teosinte but not previously reported are listed in S3 Table. Details on how these traits were measured are summarized here. (1) BRAN: Number of visible lateral branches along the main culm; counted visually. (2) CULM: Diameter of the main culm just above the ground level measured in plane with the leaves (narrowest dimension); measured using a caliper. (3) FCLN: Average length of 10-50 fruitcases along the longest apical-basal axis toward the glume side of the fruitcase; measured using SmartGrain [7]. (4) FCLW: Ratio of fruitcase length to width; derived from FCLN/FCWD. FCWD is equivalent to Ear Diameter (ED). (5) FCTR: Ratio of actual area of a fruitcase in profile to theoretical area as given by (FCLN x FCWD)/2, larger value means less triangular; derived from (actual area)/(theoretical area). (6) SDDM: Germination tests were performed on harvested teosinte fruitcases. Twenty normal fruitcases were split into two replicates of ten fruitcases each and processed in parallel. Each set of the fruitcases was germinated on a 15 x 20 cm piece of brown germination paper (Anchor Paper Co., Saint Paul, MN) that was previously wetted in a tray of deionized water. Then, the ten fruitcases were spread out on the paper and the paper was rolled up so that the fruitcases were securely but not tightly held in the roll. Twenty-five such rolls were arranged on a sheet of aluminum foil in two layers with a piece of wetted and squeezed sponge paper between the layers. The foil was folded and rolled to seal the moisture within the foil wrap. The finished wraps were placed in two incubation chambers, such that the replicates were tested in similar but independent environments. The incubation process was maintained at 37°C and complete darkness for five days. Five days after “planting” the fruitcases, the wraps were removed for scoring of the germination rate. Each fruitcase was examined separately and counted as germinated if a shoot or root tip had visibly emerged. Germination rates were recorded as a percentage germinated of the ten fruitcases in the roll. Finally, the germination rates from the two replicates were averaged. (7)

STAM: Percentage of male spikelets in the terminal inflorescence of the 2nd or 3rd lateral branch from the top of the plant; scored by visual approximation.

Genome-Wide Association Study (GWAS)

Given that our populations contain related individuals (sibling and half sibs) with high linkage disequilibrium (LD), standard GWAS analysis intended for random mating populations is inappropriate. Therefore, we implemented a scan of the genome using stepwise regression analysis. However, because stepwise regression is computationally demanding, it was not feasible to fit models with all 17.8 or 18.9 million SNPs. To reduce SNP number, we first fit a general linear model (GLM) with field variables, the inbreeding coefficient, and the first 50 principal components (PCs) based on the GBS SNPs as covariates using *FixedEffectLMPlugin* function in TASSEL5 [6]. Principal components analysis (PCA) was performed using GBS markers with the *PrincipalComponentsPlugin* function in TASSEL5 [6]. We chose to use 50 PCs, given that we had 49 teosinte and 40 landrace parents (families), and thus 50 PCs should capture most population structure attributable to individual parents (or families). To test whether the PCs were accounting for significant family effects, we used the *lm* function in R to perform linear regression for each PC by each trait in both teosinte and landrace. Almost all of these regression analyses were significant, indicating that the PCs were capturing variation attributable to family effects. The final GLM model was:

$$Y_{ij} = \mu + E_i + (F_{ij} - \bar{F}_{..})\beta_F + (F_{ij} - \bar{F}_{i.})\beta_{Fi} + x_{Sij}\beta_S + x_{Bij}B(Y)_i + x_{Rij}\beta_{R1i} + x_{Rij}^2\beta_{R2i} + x_{Rij}^3\beta_{R3i} + x_{Rij}^4\beta_{R4i} + x_{Cij}\beta_{C1i} + x_{Cij}^2\beta_{C2i} + x_{Cij}^3\beta_{C3i} + x_{Rij}^4\beta_{C4i} + PC1_{ij}\beta_{PC1} + PC2_{ij}\beta_{PC2} + \cdots + PC50_{ij}\beta_{PC50} + x_{Aij}\beta_A + x_{Dij}\beta_D + \varepsilon_{ij},$$

Where:

Y_{ij} is the observed phenotype on individual *j* in environment *i*.

The following fixed effects are included in the model:

E_i is the effect of environment (year) *i*;

F_{ij} is the marker-based inbreeding coefficient estimate for individual *j* in environment *i;*

$\bar{F}_{..}$ is the average inbreeding coefficient for all individuals across both years;

$\bar{F}_{i.}$ is the mean inbreeding coefficient for all individuals in environment *i*;

β_F is the average regression coefficient for phenotypes on the inbreeding coefficient;

β_{Fi} is the interaction effect of inbreeding depression effect with years;

x_{Sij} is the deviation of the shading measurement on the *ij*th individual from the overall average shading measurement;

β_S is the average shading effect;

x_{Bij} is a dummy variable indicating if a plant is in an edge (border) row for teosinte plants or in a row adjacent to a tractor tire passing lane for maize landrace plants;

$B(Y)_i$ is the effect of border rows in the first year (since no plants were measured in border rows in the second year);

x_{Rij}^p and x_{Cij}^p are *p* = first to fourth order polynomials of the deviation in the row and column directions, respectively, of the *ij*th plant's position from the center of the field in year *i*;

β_{Rpi} and β_{Cpi} are the regression coefficients associated with the *p*th polynomials for row and column trend effects within year *i*, respectively;

PCn_{ij} is n = 1 to 50 PCs of the *ij*th plant accounting for family background;

β_{PCn} is the average effect associated with the *n*th PCs;

x_{Aij} is a dummy variable indicating the number of minor alleles (0,1,2) at the marker for the *ij*th individual;

β_A is the additive genetic effect estimate for the marker;

x_{Dij} is a dummy variable indicating if the marker is homozygous or heterozygous for the *ij*th individual;

β_D is the dominance genetic effect estimate for the marker;

ε_{ij} is the intercept associated with the *ij*th plant.

We then selected the SNP with the lowest P value within each 200-SNP bin along each chromosome, a process that resulted in about 0.8 million SNPs each for teosinte and landrace. This set of SNPs was used for stepwise regression to map quantitative trait loci (QTLs) for each trait. We fit an additive plus dominance model with the same covariates as used for GLM with the *StepwiseAdditiveModelFitterPlugin* function in TASSEL5 [6]. For shading effect, we divided the original units by 100 as the original values for shading were out of range for the software and produced output without P values. The additive genotype scores were coded as 2 for homozygous major genotype, 1 for heterozygous genotype, and 0 for homozygous minor genotype. Since stepwise regression does not allow missing genotypic data, we imputed missing genotypes by setting them equal to the mean of the genotypic scores for all non-missing taxa. The dominance score was computed as 1 – abs(a – 1) for non-missing genotypes. For missing genotypes, we set the dominance score to 0 as imputation may cause overestimation of dominance effects. The P value to enter and leave the stepwise regression model was determined separately for each trait by empirical estimation of the genome-wise α=0.05 Type I error rate from genome scans of 1000 permutations of the trait data with respect to the independent variables. We then calculated the standardized additive effect as |A|/σ for each QTL, where A is additive effect and σ is standard deviation.

Variance Component Analysis (VCA)

To estimate the proportion of trait additive genetic variation associated with different classes of SNPs, we used a procedure to estimate variance components associated with different subsets of the SNPs [8-11]. In brief, variance component analysis (VCA) was done by (1) classifying SNPs into subsets based on a hypothesis of interest, (2) generating kinship matrices for each subset using TASSEL5 [6], and (3) fitting these kinship matrices along with phenotypic data into LDAK5 (http://dougspeed.com/ldak/), using a generalized restricted maximum likelihood (REML) solver to partition the genetic variance (heritability, h^2) into the proportion accounted for by each SNP subset [9, 10].

We grouped SNPs in two ways for VCA. First, we grouped SNPs by recombination rate. We counted the number of crossovers among progeny within 10 kb windows along the chromosomes and used these counts to calculate the recombination rate in centimorgan (cM) per 10 kb. The recombination rate for each window was assigned to all SNPs in that window. We also counted the number of genes in each window and the 10-kb recombination rates after accounting for gene number were then sorted and partitioned into five quintiles for calculation of five kinship matrices. The kinship matrices were then fit into a mixed model to simultaneously estimate the additive variances associated with each kinship matrix for both teosinte and landrace. The additive variances across 18 traits were averaged and plotted.

Selection is less effective in regions of low recombination, which is known as the Hill-Robertson effect. This effect can produce a loss of genetic variance in regions of low recombination following a selective episode. Reduction in genetic variance in regions of low recombination can also be caused by the Bulmer effect, when selection generates negative linkage equilibrium (LD), which reduces the additive variance. To compare the LD structures within the regions of different recombination rate, we calculated pairwise LD between SNPs in parents using Plink v1.90 (https://www.cog-genomics.org/plink2/) with defaulted parameters.

Second, we grouped SNPs by F_{ST} between teosinte and maize landrace. F_{ST} was calculated according the method of Weir and Cockerham [12]. F_{ST} between teosinte and maize landrace was calculated for each SNP separately using VCFtools [13] and then averaged for non-overlapping bins of 50 SNPs along each chromosome. The average value for each bin was assigned to all SNPs in that bin. The F_{ST} values were then sorted and divided into five quintiles for calculation of five kinship matrices. The estimation of F_{ST} used all 17.8 million SNPs segregating in teosinte and all 18.9 million SNPs segregating in landrace, 32.5 million total SNPs. The calculation of the five kinship matrices for teosinte and landrace were based only on those SNPs segregating in teosinte or landrace, respectively. The kinship matrices were then fit into a mixed model to simultaneously estimate the additive variances associated with each kinship matrix for both teosinte and landrace. The additive variances across 18 traits were

averaged and plotted. We also calculated Watterson's θ to compare effective population size using VariScan software (http://www.ub.edu/softevol/variscan/) by 10-kb non-overlapping windows with the common set of 4.2 million SNPs.

References

1. Yang CJ, Samayoa LF, Bradbury PJ, Olukolu BA, Xue W, York AM, et al. The genetic architecture of teosinte catalyzed and constrained maize domestication. Proc Natl Acad Sci USA. 2019; 116: 5643–5652.
2. Saghai-Maroof MA, Soliman KM, Jorgensen RA, Allard RWL. Ribosomal DNA spacer-length polymorphisms in barley: Mendelian inheritance, chromosomal location, and population dynamics. Proc Natl Acad Sci USA. 1984; 81: 8014–8018.
3. Jiao Y, Peluso P, Shi J, Liang T, Stitzer MC, Wang B, et al. Improved maize reference genome with single-molecule technologies. Nature. 2017; 546: 524.
4. Li H. Aligning sequence reads, clone sequences and assembly contigs with BWA-MEM. arXiv: 1303.3997v2 [Preprint]. 2013 [cited 2020 Feb 10]: [3 p.]. Available from: https://arxiv.org/abs/1303.3997
5. Freed DN, Aldana R, Weber JA, Edwards JS. (2017) The Sentieon Genomics Tools - A fast and accurate solution to variant calling from next-generation sequence data. BioRxiv [Preprint]. 2017 bioRxiv 115717 [posted 2017 Mar 10; revised 2017 Mar 12; cited 2020 Feb 10]: [11 p.]. Available from: https://www.biorxiv.org/content/10.1101/115717v2 doi: https://doi.org/10.1101/115717
6. Bradbury PJ, Zhang Z, Kroon DE, Casstevens TM, Ramdoss Y, Buckler ES. TASSEL: software for association mapping of complex traits in diverse samples. Bioinformatics. 2007; 23: 2633–2635.
7. Tanabata T, Shibaya T, Hori K, Ebana K, Yano M. SmartGrain: high-throughput phenotyping software for measuring seed shape through image analysis. Plant Physiol. 2012; 160:1871–1880.
8. Rodgers-Melnick E, Vera DL, Bass HW, Buckler ES. Open chromatin reveals the functional maize genome. Proc Natl Acad Sci USA. 2016; 113: E3177–E3184.
9. Speed D, Balding DJ. MultiBLUP: improved SNP-based prediction for complex traits. Genome Res. 2014; 24: 1550–1557.
10. Speed D, Hemani G, Johnson MR, Balding DJ. Improved heritability estimation from genome-wide SNPs. Am J Hum Genet. 2012; 91: 1011–1021.
11. Xue S, Bradbury PJ, Casstevens T, Holland JB. Genetic architecture of domestication-related traits in maize. Genetics. 2016; 204: 99–113.
12. Weir BS, Cockerham CC. Estimating F-statistics for the analysis of population structure. Evolution. 1984; 38: 1358–1370.
13. Danecek P, Auton A, Abecasis G, Albers CA, Banks E, DePristo MA, et al. The variant call format and VCFtools. Bioinformatics. 2011; 27: 2156–2158.
