# Supplementary material for: The genetic architecture of the maize progenitor, teosinte, and how it was altered during maize domestication
Source: PLoS Genet. 2020 May 14;16(5):e1008791. doi: 10.1371/journal.pgen.1008791 (PMC7266358; doi:10.1371/journal.pgen.1008791)
Supplement: S1 Fig — (PDF) [file pgen.1008791.s002.pdf]

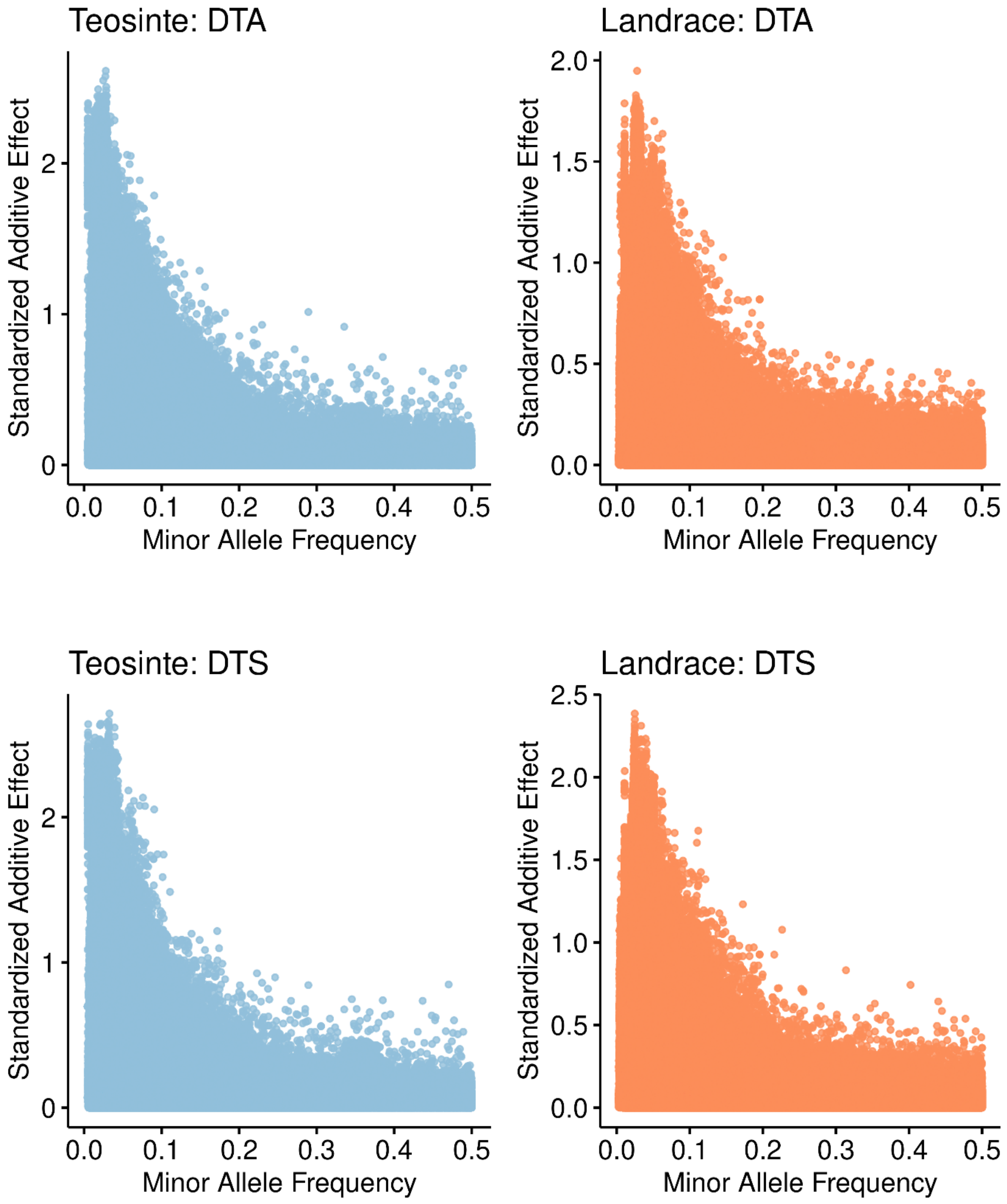

Teosinte: DTA
Landrace: DTA
Teosinte: DTS
Landrace: DTS
Standardized Additive Effect
Minor Allele Frequency
0
1
2
0.0
0.5
1.0
1.5
2.0
2.5
0.1
0.2
0.3
0.4

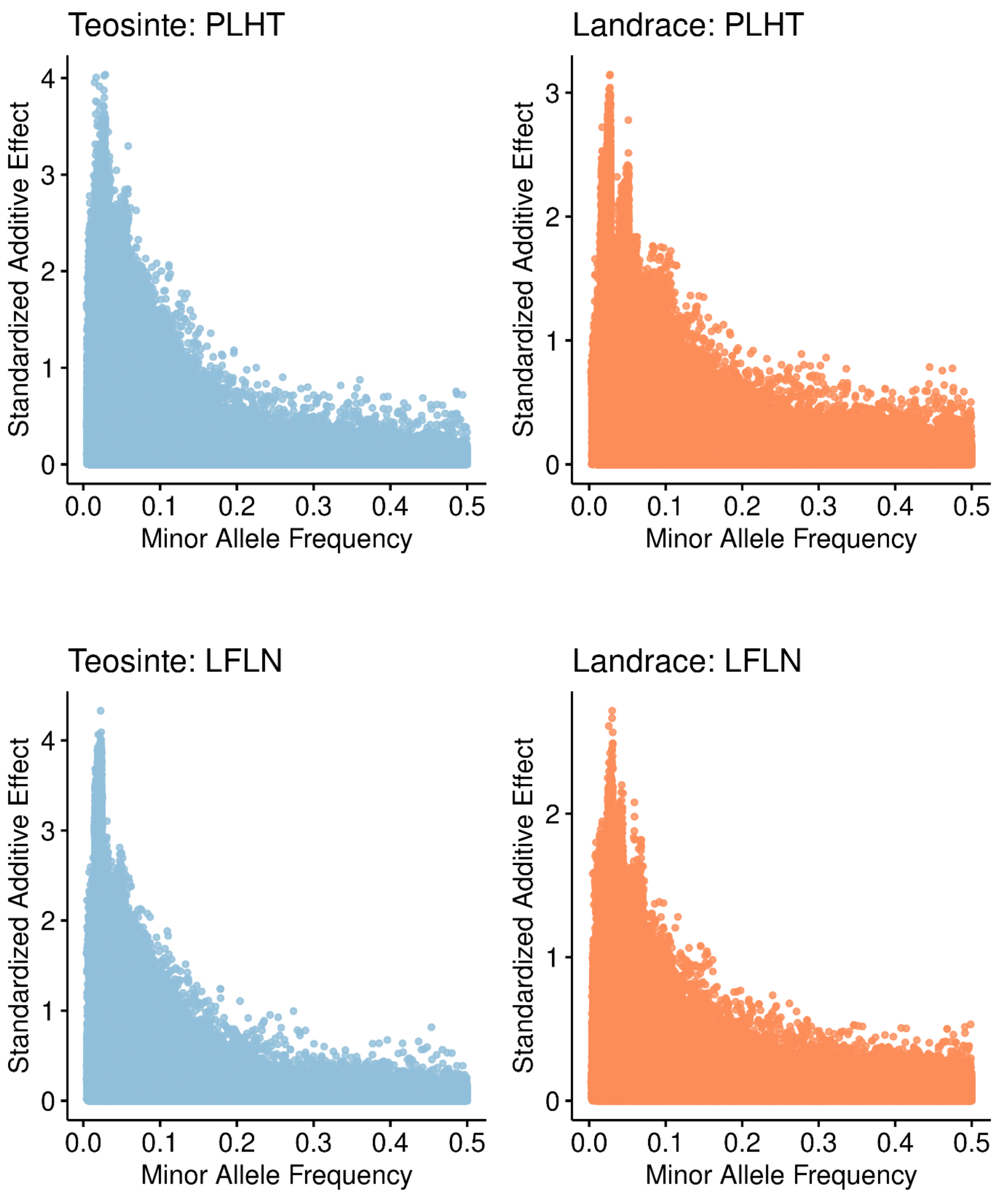

Teosinte: PLHT
Landrace: PLHT
Teosinte: LFLN
Landrace: LFLN
Standardized Additive Effect
Minor Allele Frequency
0.0
0.1
0.2
0.3
0.4
0.5
0
1
2
3
4

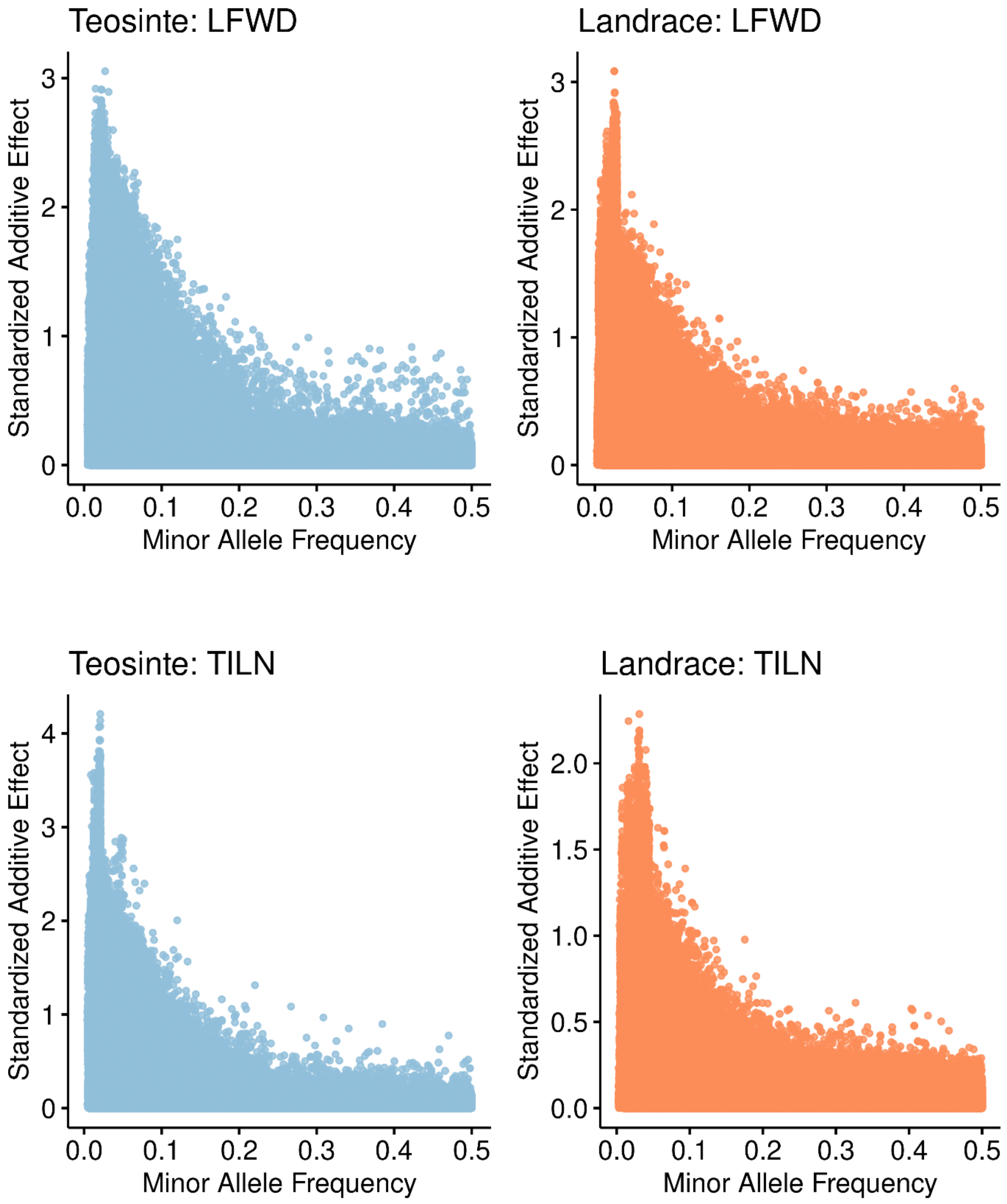

Teosinte: LFWD
Landrace: LFWD
Teosinte: TILN
Landrace: TILN
Standardized Additive Effect
Minor Allele Frequency
0.0
0.1
0.2
0.3
0.4
0.5
0
1
2
3
4
0.0
0.5
1.0
1.5
2.0

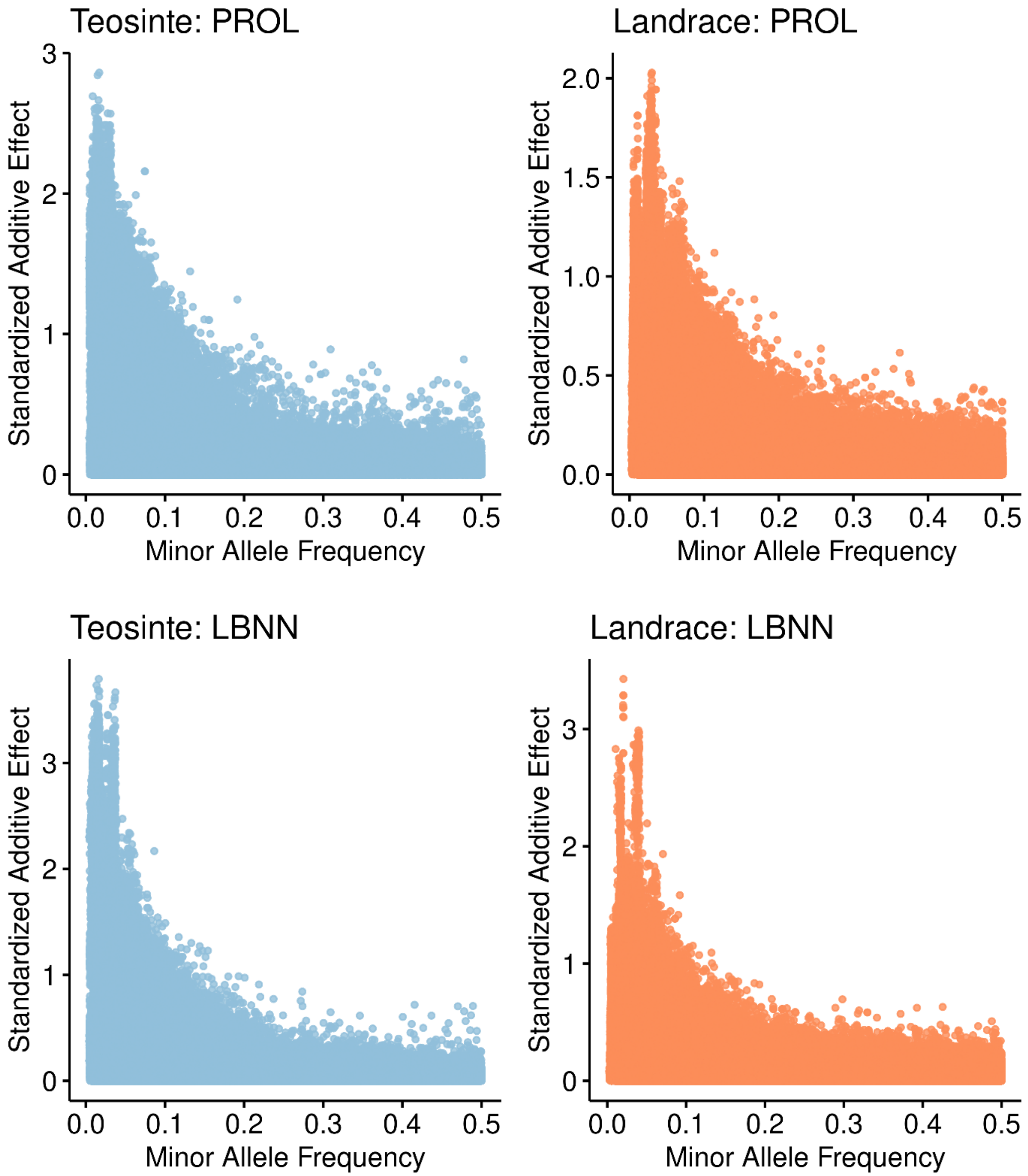

Teosinte: PROL
Landrace: PROL
Teosinte: LBNN
Landrace: LBNN
Standardized Additive Effect
Minor Allele Frequency
0.0
0.1
0.2
0.3
0.4
0.5
0
1
2
3
0.5
1.0
1.5
2.0

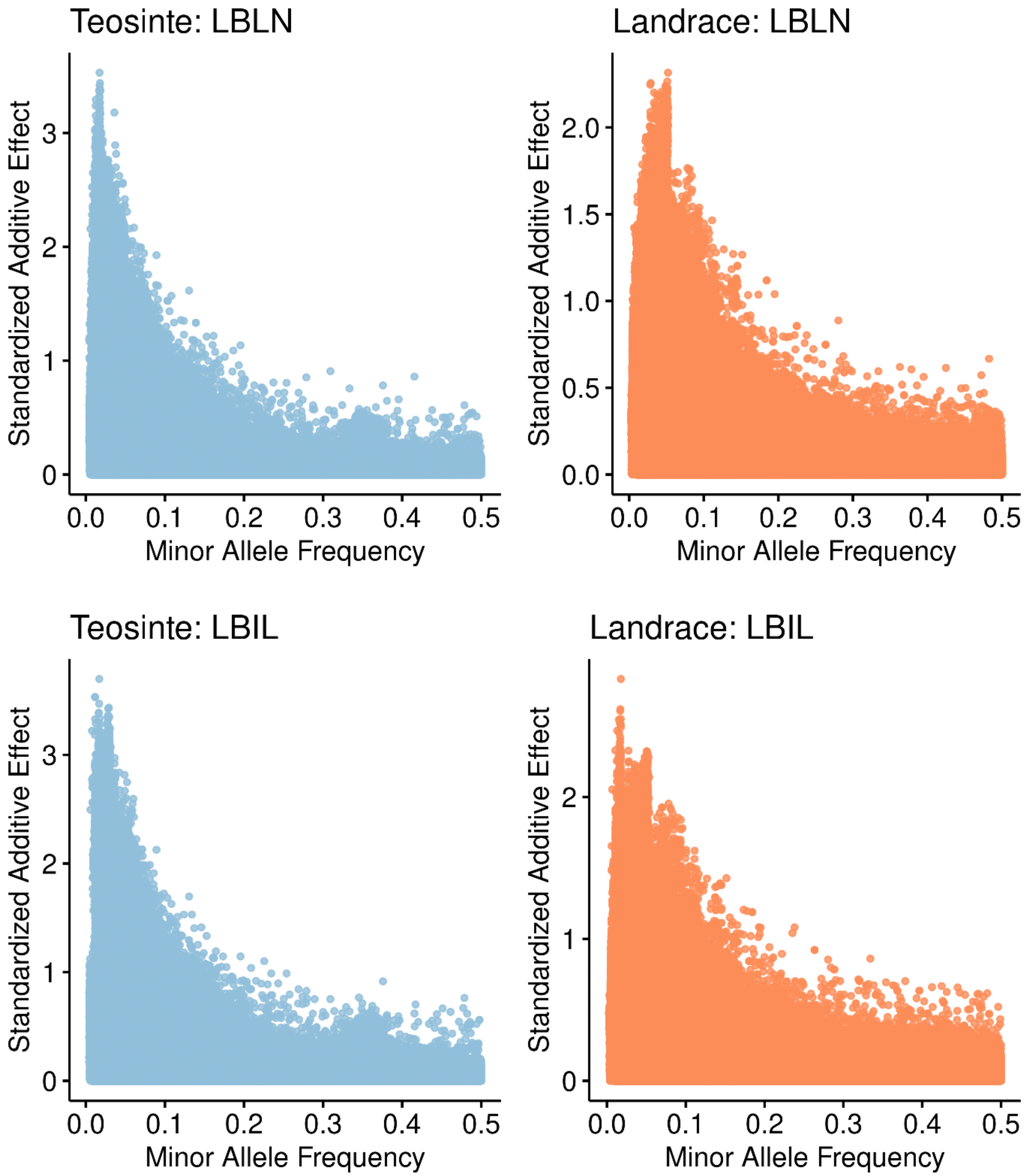

Teosinte: LBLN
Landrace: LBLN
Teosinte: LBIL
Landrace: LBIL
Standardized Additive Effect
Minor Allele Frequency
0.0
0.1
0.2
0.3
0.4
0.5
0
1
2
3
0.5
1.0
1.5
2.0

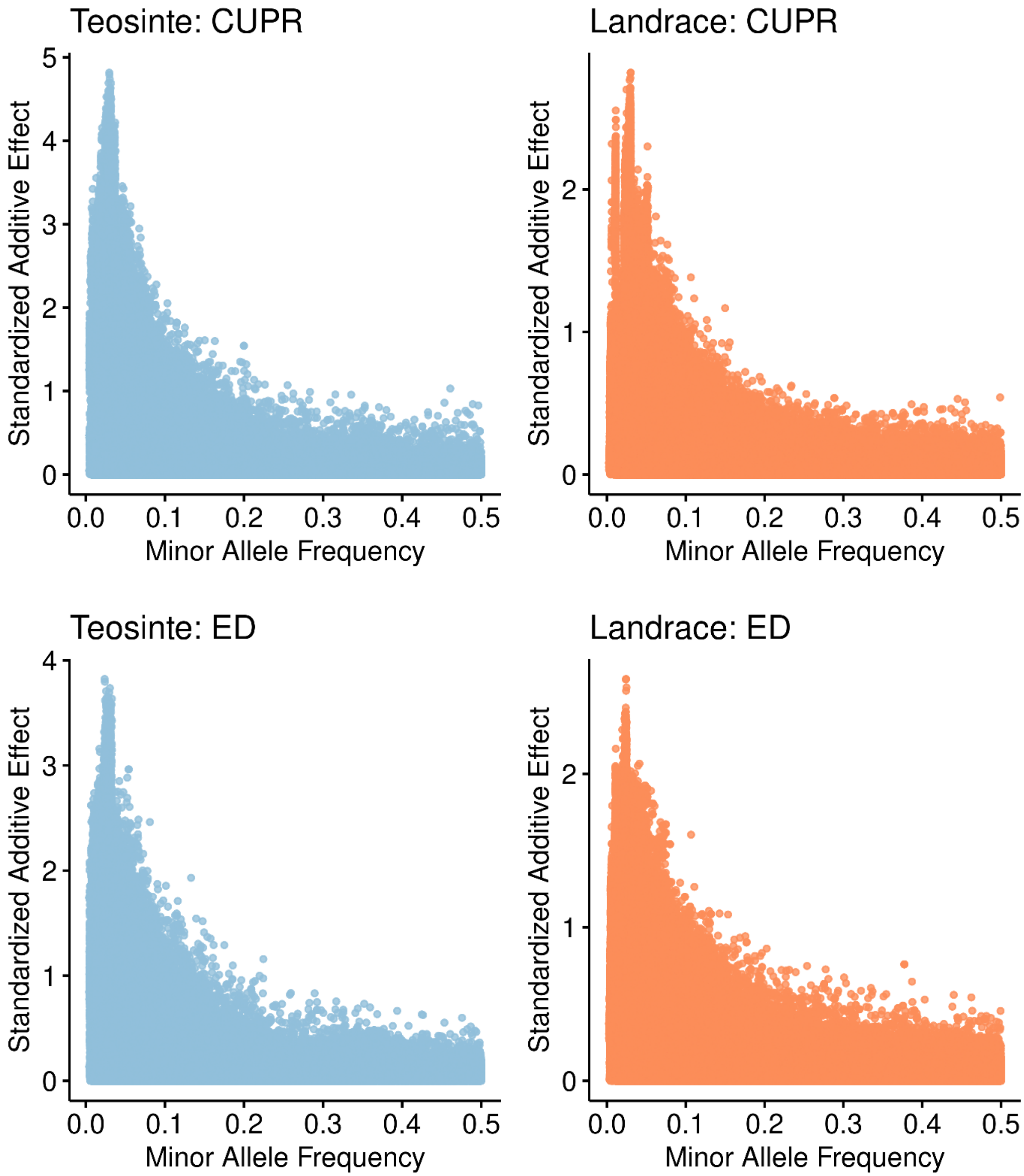

Teosinte: CUPR
Landrace: CUPR
Teosinte: ED
Landrace: ED
Standardized Additive Effect
Minor Allele Frequency
0.0
0.1
0.2
0.3
0.4
0.5
0
1
2
3
4
5

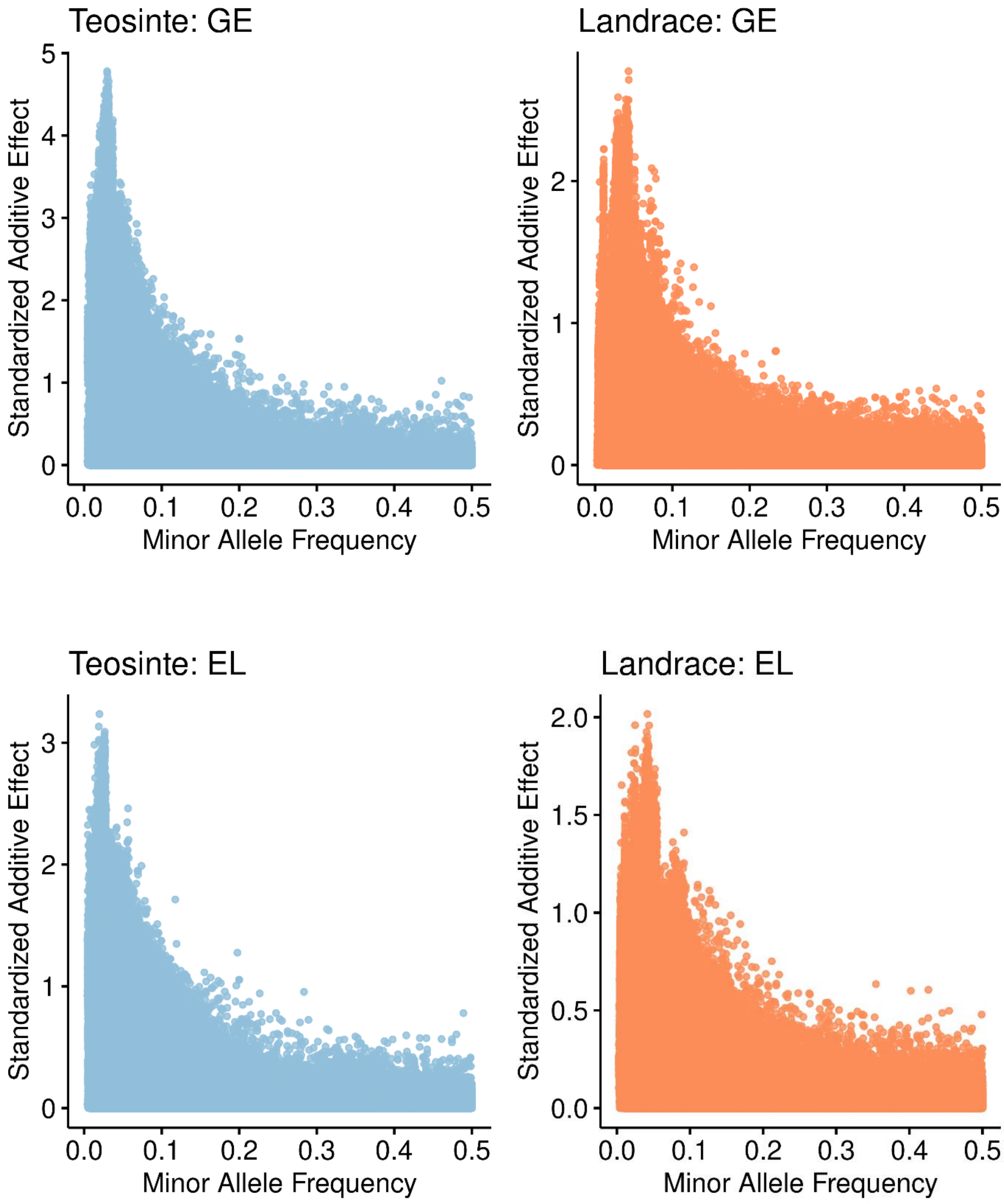
Teosinte: GE
Landrace: GE
Teosinte: EL
Landrace: EL
Standardized Additive Effect
Minor Allele Frequency
0
1
2
3
4
5
0.0
0.5
1.0
1.5
2.0
0.1
0.2
0.3
0.4
0.5

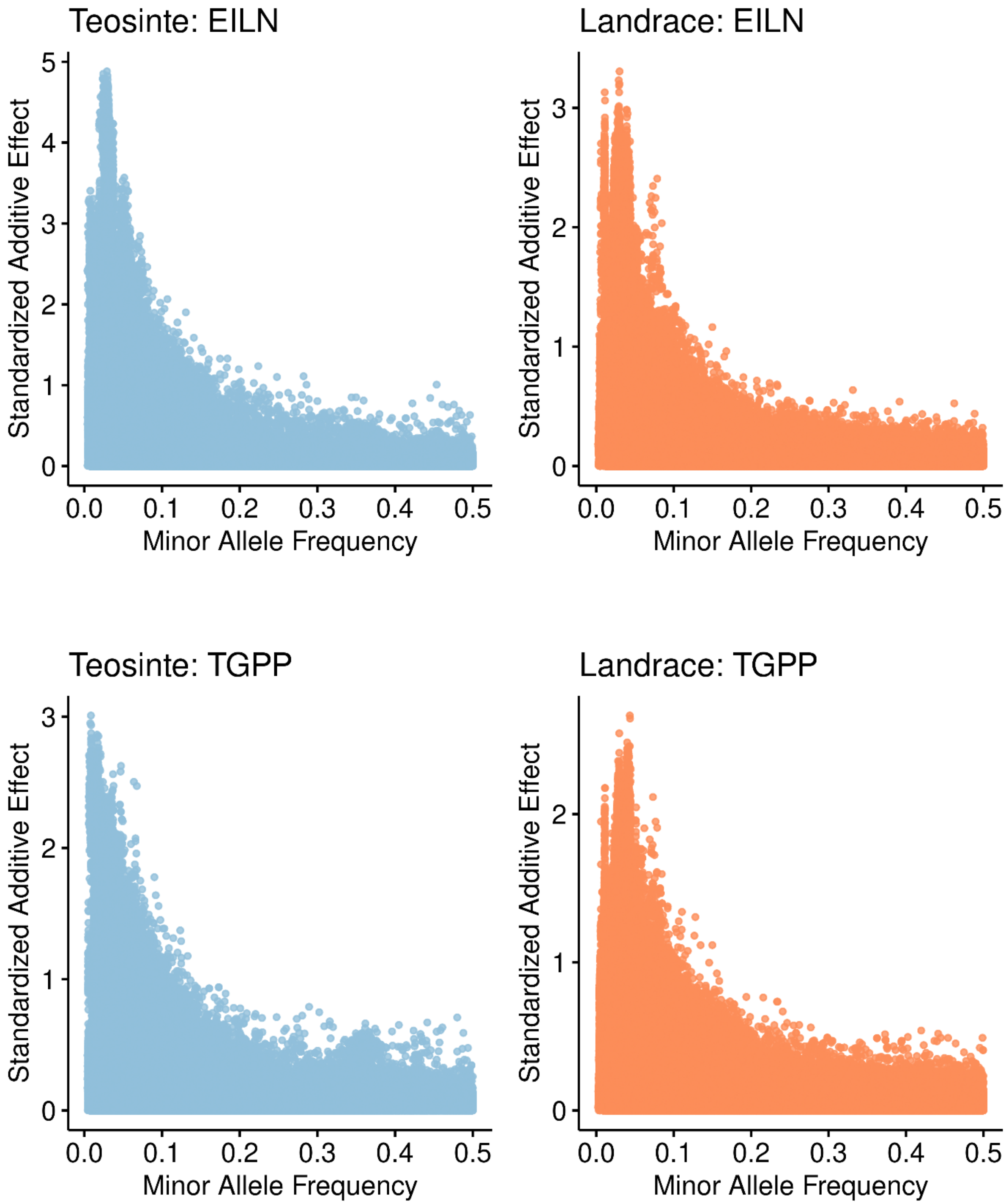
Teosinte: EILN
Landrace: EILN
Teosinte: TGPP
Landrace: TGPP
Standardized Additive Effect
Minor Allele Frequency
0.0
0.1
0.2
0.3
0.4
0.5
0
1
2
3
4
5

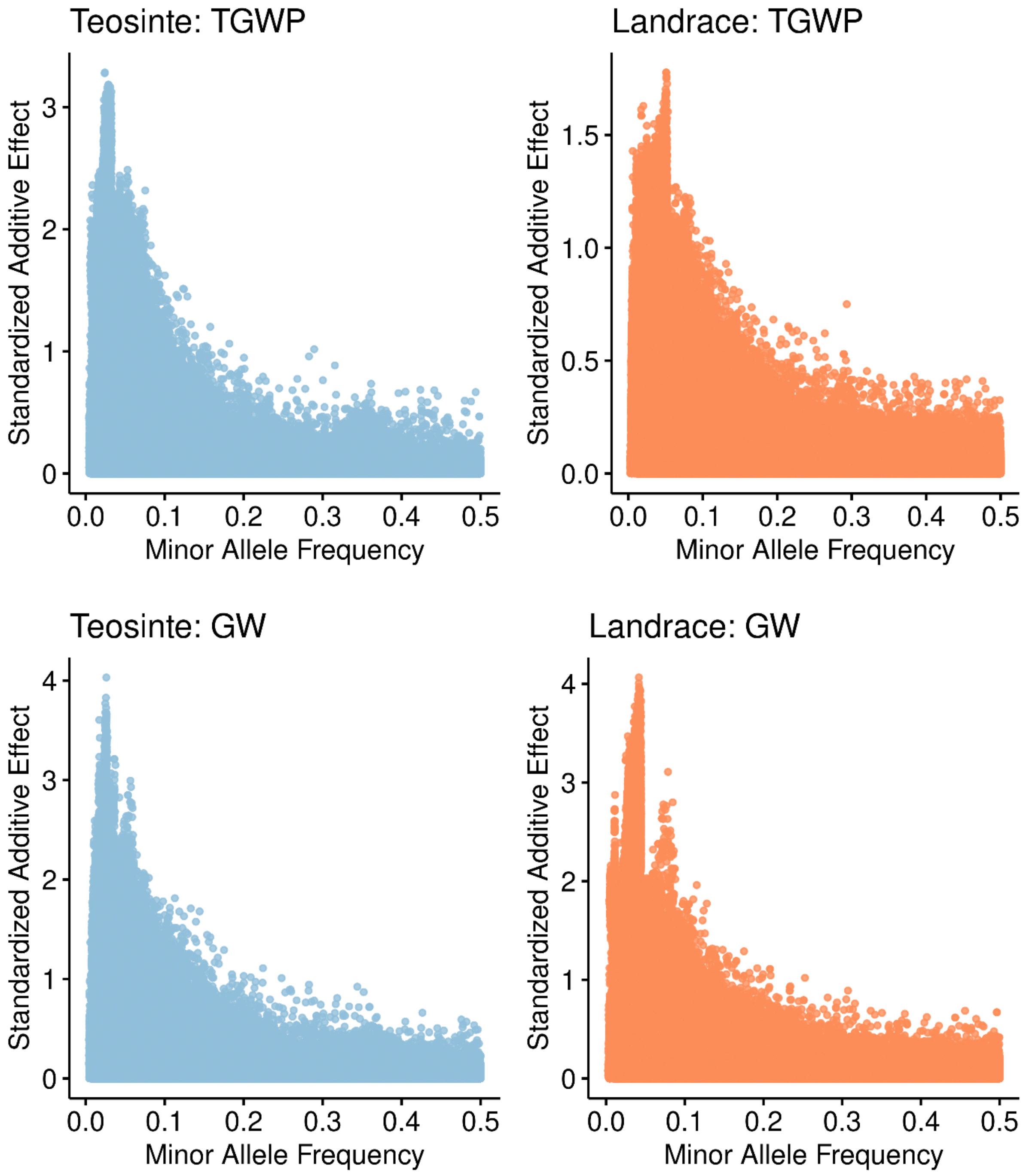

S1 Fig. The relationship between effect size and MAF for all SNPs in 18 traits.

The additive effects were estimated from the GLM model for each trait. The standardized additive effect, calculated as additive effect by phenotypic standard deviation in absolute value, is plotted against MAF for each QTL. Supporting Fig 2.
