# Supplementary material for: The genetic architecture of the maize progenitor, teosinte, and how it was altered during maize domestication
Source: PLoS Genet. 2020 May 14;16(5):e1008791. doi: 10.1371/journal.pgen.1008791 (PMC7266358; doi:10.1371/journal.pgen.1008791)
Supplement: S2 Fig — (PDF) [file pgen.1008791.s003.pdf]

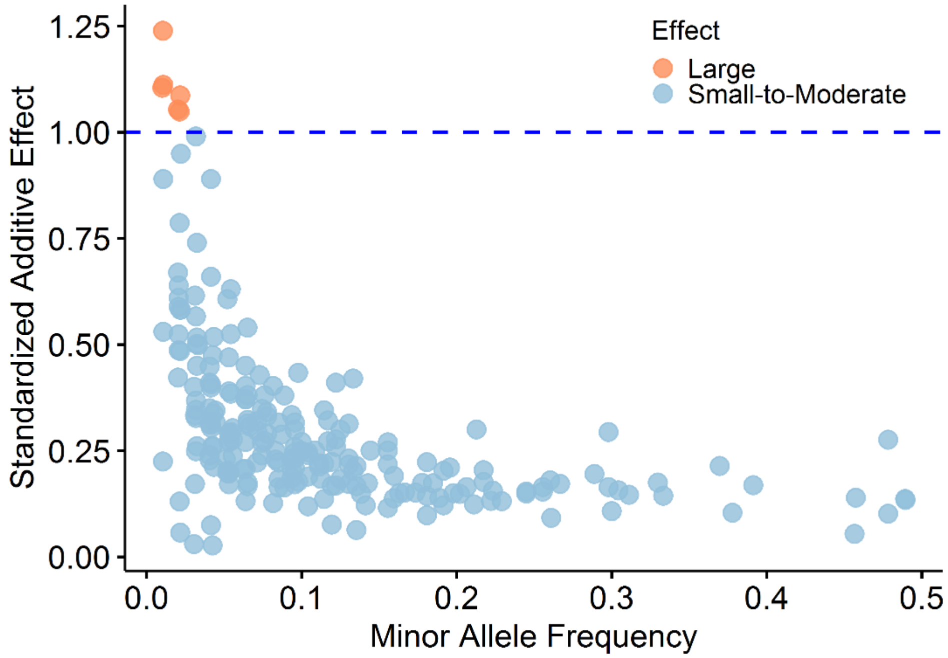

S2 Fig. The relationship between effect size and MAF for 222 QTL in seven teosinte-only traits.

The standardized additive effect, calculated as additive effect by phenotypic standard deviation in absolute value, is plotted against MAF for each QTL. MAF was calculated from parent data. Large effect QTL is defined as a QTL with a standardized additive effect greater than 1 phenotypic standard deviation as indicated by blue dotted lines.
