# Supplementary material for: The genetic architecture of the maize progenitor, teosinte, and how it was altered during maize domestication
Source: PLoS Genet. 2020 May 14;16(5):e1008791. doi: 10.1371/journal.pgen.1008791 (PMC7266358; doi:10.1371/journal.pgen.1008791)
Supplement: S3 Fig — (PDF) [file pgen.1008791.s004.pdf]

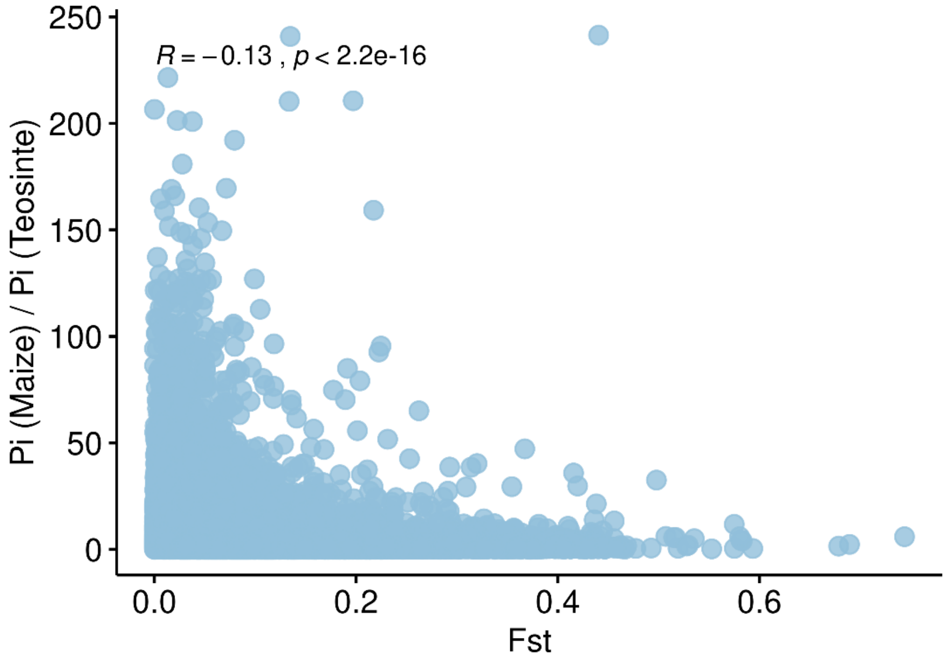

S3 Fig. The relationship between nucleotide diversity and F_{ST}.

Negative correlation is observed with a significant correlative coefficient R=-0.13 (p<2.2e-16).
