# Supplementary material for: The genetic architecture of the maize progenitor, teosinte, and how it was altered during maize domestication
Source: PLoS Genet. 2020 May 14;16(5):e1008791. doi: 10.1371/journal.pgen.1008791 (PMC7266358; doi:10.1371/journal.pgen.1008791)
Supplement: S2 Table — (PDF) [file pgen.1008791.s006.pdf]

S2 Table. Summary of heritability.

Population	Trait	h^2	QTL+PCs	QTL alone	PCs alone	QTLs and PCs share	Non-QTL	Undetected	QTL/ h^2	Non-QTL/h^2	PCs/ h^2	Undetected/h^2	No. QTL
Teosinte	DTA	0.408	0.287	0.105	0.133	0.049	0.303	0.121	0.257	0.743	0.326	0.297	29
Teosinte	DTS	0.483	0.295	0.120	0.131	0.044	0.363	0.188	0.248	0.752	0.271	0.389	31
Teosinte	PLHT	0.362	0.284	0.101	0.129	0.054	0.261	0.078	0.280	0.720	0.356	0.217	30
Teosinte	LFLN	0.255	0.241	0.060	0.187	-0.006	0.195	0.014	0.236	0.764	0.733	0.056	13
Teosinte	LFWD	0.400	0.373	0.117	0.218	0.039	0.283	0.027	0.291	0.709	0.545	0.066	30
Teosinte	EL	0.523	0.457	0.135	0.257	0.065	0.388	0.066	0.258	0.742	0.491	0.126	38
Teosinte	CUPR	0.551	0.478	0.156	0.309	0.013	0.395	0.073	0.282	0.718	0.561	0.132	37
Teosinte	ED	0.692	0.593	0.167	0.322	0.104	0.525	0.099	0.242	0.758	0.465	0.142	39
Teosinte	GE	0.551	0.478	0.156	0.309	0.013	0.395	0.073	0.282	0.718	0.561	0.132	37
Teosinte	GW	0.725	0.627	0.186	0.209	0.233	0.539	0.098	0.256	0.744	0.288	0.135	52
								**0.084**	**0.263**	**0.737**	**0.460**	**0.169**	**33.6**
Landrace	DTA	0.282	0.213	0.048	0.128	0.038	0.234	0.069	0.168	0.832	0.454	0.244	16
Landrace	DTS	0.253	0.157	0.054	0.080	0.024	0.199	0.096	0.213	0.787	0.316	0.378	11
Landrace	PLHT	0.212	0.131	0.033	0.065	0.033	0.179	0.081	0.154	0.846	0.307	0.383	12
Landrace	LFLN	0.383	0.284	0.088	0.195	0.001	0.295	0.099	0.229	0.771	0.509	0.259	23
Landrace	LFWD	0.350	0.281	0.079	0.147	0.055	0.271	0.069	0.225	0.775	0.420	0.197	27
Landrace	EL	0.157	0.112	0.036	0.077	-0.001	0.121	0.045	0.228	0.772	0.490	0.286	13
Landrace	CUPR	0.156	0.110	0.025	0.094	-0.010	0.131	0.046	0.163	0.837	0.603	0.295	8
Landrace	ED	0.261	0.224	0.016	0.206	0.001	0.245	0.037	0.063	0.937	0.789	0.141	6
Landrace	GE	0.116	0.088	0.043	0.057	-0.012	0.073	0.028	0.367	0.633	0.491	0.241	10
Landrace	GW	0.161	0.141	0.046	0.123	-0.028	0.115	0.020	0.286	0.714	0.764	0.125	6
								**0.059**	**0.210**	**0.790**	**0.514**	**0.255**	**13.2**

h^2 is calculated in Yang et al. (2019). Non-QTL= h^2 – QTL alone; Undetected= h^2 – (QTL+PCs)
