# Supplementary material for: The genetic architecture of the maize progenitor, teosinte, and how it was altered during maize domestication
Source: PLoS Genet. 2020 May 14;16(5):e1008791. doi: 10.1371/journal.pgen.1008791 (PMC7266358; doi:10.1371/journal.pgen.1008791)
Supplement: S3 Table — (PDF) [file pgen.1008791.s007.pdf]

S3 Table. Descriptions for seven teosinte-only traits analyzed in this study.

Full Name	Trait	Units	Description
Branch Number	BRAN	count	Number of visible lateral branches along the main culm.
Culm Diameter	CULM	cm	Diameter of the main culm.
Fruitcase Length	FCLN	mm	Average length of 10-50 fruitcases.
Fruitcase Length to Width Ratio	FCLW	ratio	Ratio of fruitcase length to width.
Fruitcase Triangularity	FCTR	ratio	Ratio of actual area of a fruitcase in profile to theoretical area as given by (FCLN x FCWD)/2.
Seed Dormancy	SDDM	percentage	Percentage of seeds that germinated
Percentage of Male Spikelets	STAM	percentage	Percentage of male spikelets in a terminal lateral inflorescence.
