# Supplementary material for: The genetic architecture of the maize progenitor, teosinte, and how it was altered during maize domestication
Source: PLoS Genet. 2020 May 14;16(5):e1008791. doi: 10.1371/journal.pgen.1008791 (PMC7266358; doi:10.1371/journal.pgen.1008791)
Supplement: S4 Table — (PDF) [file pgen.1008791.s008.pdf]

**S4 Table. Comparison of LD for regions of different recombination rate.**

| Population | Category | Average LD (r^2) | p-value (t test) |
|---|---|---|---|
| Teosinte | Qu20 | 0.645 | |
| | Qu40 | 0.637 | 0 |
| | Qu60 | 0.636 | 0 |
| | Qu80 | 0.631 | 0 |
| | Qu100 | 0.625 | 0 |
| Landrace | Qu20 | 0.720 | |
| | Qu40 | 0.724 | 9.99E-208 |
| | Qu60 | 0.719 | 8.12E-14 |
| | Qu80 | 0.716 | 5.04E-164 |
| | Qu100 | 0.698 | 0 |

Qu20, Qu40, Qu60, Qu80 and Qu100 are quintiles of the 10 kb recombination rates after accounting for gene density. t tests were performed in contrast to Qu20.
