# Supplementary material for: The genetic architecture of the maize progenitor, teosinte, and how it was altered during maize domestication
Source: PLoS Genet. 2020 May 14;16(5):e1008791. doi: 10.1371/journal.pgen.1008791 (PMC7266358; doi:10.1371/journal.pgen.1008791)
Supplement: S5 Table — (PDF) [file pgen.1008791.s009.pdf]

S5 Table. Number of progeny used for phasing teosinte parents.

		Dad																																														
		PC_I05_ID1	PC_I06_ID1	PC_I08_ID1	PC_I11_ID2	PC_I50_ID1	PC_I50_ID2	PC_I52_ID1	PC_I55_ID2	PC_I58_ID1	PC_I58_ID2	PC_J04_ID1	PC_J07_ID2	PC_J10_ID1	PC_J12_ID1	PC_J13_ID1	PC_J14_ID2	PC_J48_ID2	PC_J50_ID1	PC_J51_ID1	PC_K02_ID1	PC_K54_ID1	PC_K55_ID2	PC_K60_ID1	PC_L06_ID2	PC_L08_ID1	PC_L12_ID1	PC_L12_ID2	PC_L14_ID1	PC_L48_ID2	PC_L56_ID1	PC_M05_ID1	PC_M15_ID1	PC_M58_ID1	PC_M59_ID1	PC_N03_ID2	PC_N04_ID1	PC_N07_ID1	PC_N07_ID2	PC_N09_ID1	PC_N10_ID2	PC_N11_ID1	PC_N13_ID1	PC_N14_ID2	PC_N48_ID1	PC_N57_ID2	PC_N58_ID2	PC_O08_ID2	PC_O51_ID2	PC_O59_ID2
Mom	PC_I05_ID1	4																																																
	PC_I06_ID1		4																																															
	PC_I08_ID1			4																																														
	PC_I11_ID2				4																		1																									1		
	PC_I50_ID1					4																																												
	PC_I50_ID2						4											1																																
	PC_I52_ID1							4																																										
	PC_I55_ID2								4																																									
	PC_I58_ID1									4																																								
	PC_I58_ID2								1		4																																							
	PC_J04_ID1											4																																						
	PC_J07_ID2												4																																	1	1			
	PC_J10_ID1													3																					14															
	PC_J12_ID1														4																																			
	PC_J13_ID1															4																																		
	PC_J14_ID2																4																																	
	PC_J48_ID2																	4																					1											
	PC_J50_ID1																		4																															
	PC_J51_ID1																			4																														
	PC_K02_ID1																				4																													
	PC_K54_ID1																					4																												
	PC_K55_ID2																						4																											1
	PC_K60_ID1																							4																										
	PC_L06_ID2								2																4																									
	PC_L08_ID1																									4																								
	PC_L12_ID1																										4																							
	PC_L12_ID2																											4																						
	PC_L14_ID1																												4																					
	PC_L48_ID2						1																							4																	1			
	PC_L56_ID1																														4																			
	PC_M05_ID1																															4																		
	PC_M15_ID1																																4																	
	PC_M58_ID1																																	4																
	PC_M59_ID1													1																					4															
	PC_N03_ID2								1									1																		4														
	PC_N04_ID1																																				4													
	PC_N07_ID1																																					4												
	PC_N07_ID2																	2																					4											
	PC_N09_ID1																																							4										
	PC_N10_ID2																																								4									
	PC_N11_ID1																																									4								
	PC_N13_ID1																																										4							
	PC_N14_ID2																																											4						
	PC_N48_ID1																																												4					
	PC_N57_ID2												1																																	4				
	PC_N58_ID2																																														4			
	PC_O08_ID2																																													1	1	4		
	PC_O51_ID2																																																4	
	PC_O59_ID2																						1																											4
