# Supplementary material for: The genetic architecture of the maize progenitor, teosinte, and how it was altered during maize domestication
Source: PLoS Genet. 2020 May 14;16(5):e1008791. doi: 10.1371/journal.pgen.1008791 (PMC7266358; doi:10.1371/journal.pgen.1008791)
Supplement: S6 Table — (PDF) [file pgen.1008791.s010.pdf]

S6 Table. Number of progeny used for phasing maize landrace parents.

		Dad																																					
		164_1	164_2	164_4	164_8	164_11	164_14	165_4	165_6	165_7	165_10	166_3	166_9	166_10	166_11	166_12	167_1	167_3	167_5	167_8	167_12	167_13	168_2	168_5	169_6	169_11	170_1	170_2	170_4	170_6	170_7	170_11	170_12	171_4	171_5	171_6	171_7	171_8	171_10	171_11	171_13
Mom	164_1	4																																							
	164_2		4																																						
	164_4			1														5																							
	164_8				4																																				
	164_11					1																														4					
	164_14						4																																		
	165_4							4							7																										
	165_6								6																																
	165_7									9																															
	165_10																																								
	166_3											4																													
	166_9												4																												
	166_10													4																											
	166_11																																								
	166_12															4																									
	167_1																																								
	167_3																	6																							
	167_5	7																																							
	167_8																			4																					
	167_12																				4																				
	167_13																					5	2																		
	168_2																																								
	168_5																							4																	
	169_6																								3																
	169_11																									4															
	170_1																										4														
	170_2																											4													
	170_4																												4												
	170_6																													4											
	170_7																														4										
	170_11																															4									
	170_12																																4								
	171_4																																	4			7				
	171_5																																		4						
	171_6										7						7																			4					
	171_7																																								
	171_8																						7															4			
	171_10																																						4		
	171_11																																							4	
	171_13																																								4
