# Supplementary material for: The genetic architecture of the maize progenitor, teosinte, and how it was altered during maize domestication
Source: PLoS Genet. 2020 May 14;16(5):e1008791. doi: 10.1371/journal.pgen.1008791 (PMC7266358; doi:10.1371/journal.pgen.1008791)
Supplement: S1 Text — (PDF) [file pgen.1008791.s011.pdf]

S1 Text. Perl script for phasing parents.

```
################################################################################ code start

#!/usr/bin/perl -w
use strict;
die usage() unless @ARGV==2;
my ($chromosome, $family)= @ARGV;

open IN1,"/mnt/sas0/AD/qchen295/C2/Phasing/Teo/Teo_family$family";
open LINE,  "/mnt/sas0/AD/qchen295/C2/Phasing/Teo/Teo_family$family";
my @sample;
while (<LINE>)  {
	chomp;
	my ($sample,$parent1,$parent2)= (split /\t/, $_)[0,1,2];
	push (@sample, $sample, $parent1, $parent2);
}
my %seen;
my @unique = grep { !$seen{$_}++ } @sample;
my $unique = join  ",", @unique;

open IN2,  "bcftools view -s $unique teo_phase1_c$chromosome\_snps.bcf | grep -v '##' | awk -f
Extract_columns_by_header.awk -v cols=#CHROM,POS,ID,REF,ALT,$unique   |";
open IN3,  "bcftools view -h teo_phase1_c$chromosome\_snps.bcf | grep '#CHROM'  | awk -f
Extract_columns_by_header.awk -v cols=#CHROM,POS,ID,REF,ALT,$unique   |";
open IN4,  "parentcalls_chr$chromosome.txt";
open (OUT,  "+>out_chr${chromosome}/mom_dad_phase_out_chr$chromosome\_family$family.txt") || die "Cannot
write to file out_chr${chromosome}/mom_dad_phase_out_chr$chromosome\_family$family.txt";

my @lines=<IN2>;
my $header=<IN3>;
my @AT=<IN4>;

my %id2range = ();
foreach my $l (@AT)
{
	chomp $l;
	my ($id,$chr,$start,$end,$geno)= split /\t/, $l;
	if ($chr eq $chromosome)
	{
		push @{$id2range{$id}},    (join "\t", ($start,$end,$geno));
	}
}

LOOP1:while (<IN1>) {
```

```
next LOOP1  unless (/\w/);
chomp;
my @info=split /\t/,$_;
my @range = ();
if (exists $id2range{$info[0]})
{
	@range = @{$id2range{$info[0]}};
}
else
{
	print "Warning: $info[0] does not have parental calls, assume homozygous\n";
	#next LOOP1;
}

if ($info[3]=~/self/) {
	my ($pos1,$pos2,$pos3)= &get_position($info[0],$info[1],$info[2],$header);
	#print "$pos1\t$pos2\t$pos3\t$info[1]\n";
	for (my $i=0;$i<@lines;$i++) {
		chomp $lines[$i];
		if ($lines[$i]!~/#/) {
			my @eles= split /\t/, $lines[$i];
			my ($kid,$mot,$fat)= ($eles[$pos1],$eles[$pos2],$eles[$pos3]);
			my $dadphase="";
			if ($mot=~/0\/1/ && $fat=~/0\/1/) {
				my $A=0;
				my $T=0;
				my $C=0;
				LOOP2:foreach (@range) {

					my ($start,$end,$geno)= split /\t/, $_;

					if (($eles[1]>=$start) && ($eles[1]<=$end))
					{
					     if ($geno eq "A")    {$A++;}
					     elsif ($geno eq "T")    {$T++;}
					     elsif ($geno eq "C")    {$C++;}
					}
				}
				if (($A + $T + $C)==0)
				{
					$dadphase = "./.";
				}
				elsif ($kid=~/0\/0/) {
					if ($C!=0) {
```

```
					$dadphase="./.";
				} elsif ($A!=0) {
					$dadphase="0|1";
				} elsif ($T!=0) {
					$dadphase="1|0";
				} else {
					$dadphase="./.";
				}
			} elsif ($kid=~/0\/1/) {
				$dadphase="./.";
			} elsif ($kid=~/1\/1/) {
				if ($C!=0) {
					$dadphase="./.";
				} elsif ($A!=0) {
					$dadphase="1|0";
				} elsif ($T!=0) {
					$dadphase="0|1";
				} else {
					$dadphase="./.";
				}
			} elsif ($kid=~/\.\/\./) {
				$dadphase="./.";
			}
			print OUT "$info[0]\t$eles[0]\t$eles[1]\t$eles[3]\t$eles[4]\t$dadphase\n";
		} elsif ($mot=~/0\/0/ && $fat=~/0\/0/) {
			print OUT "$info[0]\t$eles[0]\t$eles[1]\t$eles[3]\t$eles[4]\t0|0\n";
		} elsif ($mot=~/1\/1/ && $fat=~/1\/1/) {
			print OUT "$info[0]\t$eles[0]\t$eles[1]\t$eles[3]\t$eles[4]\t1|1\n";
		} elsif ($mot=~/.\/./ && $fat=~/.\/./) {
			print OUT "$info[0]\t$eles[0]\t$eles[1]\t$eles[3]\t$eles[4]\t.\/.\n";
		}
	}
}
} elsif ($info[3]=~/outcross/) {
	my ($pos1,$pos2,$pos3)=&get_position($info[0],$info[1],$info[2],$header);
	#print "$pos1\t$pos2\t$pos3\t$info[1]\n";
	for (my $i=0;$i<@lines;$i++) {
		chomp $lines[$i];
		if ($lines[$i]!~/#/) {
			my @eles= split /\t/, $lines[$i];
			my ($kid,$mot,$fat)= ($eles[$pos1],$eles[$pos2],$eles[$pos3]);
			my @t = split ":", $kid; $kid = $t[0];
			my $dadphase="";
			my $momphase="";
```

```
my %parentalCalls = ();
foreach (@range) {
	my ($start,$end,$geno)= split /\t/, $_;
	if (($eles[1]>=$start) && ($eles[1]<=$end))
	{
		$parentalCalls{$geno} ++;
	}
}

# get the concensus call in the region
my @sorted = reverse sort {$parentalCalls{$a}<=>$parentalCalls{$b}} (keys %parentalCalls);
my $parentalCall = $sorted[0];

if (!(defined $parentalCall))
{
	$dadphase= "./.";
	$momphase= "./.";
}
elsif ($mot=~/0\/0/ && $fat=~/0\/1/) {
	$momphase= "0|0";
	if (($kid=~/1/) && ($parentalCall=~/A|G/))
	{
		$dadphase= "1|0";
	}
	elsif (($kid=~/1/) && ($parentalCall=~/T|C/))
	{
		$dadphase= "0|1";
	}
	else {
		$dadphase= "./.";
	}
} elsif ($mot=~/1\/1/ && $fat=~/0\/1/) {
	$momphase= "1|1";
	if (($kid=~/0/) && ($parentalCall=~/A|G/))
	{
		$dadphase= "0|1";
	}
	elsif (($kid=~/0/) && ($parentalCall=~/T|C/))
	{
		$dadphase= "1|0";
	}
	else {
		$dadphase= "./.";
	}
```

```
} elsif ($mot=~/0\/1/ && $fat=~/0\/0/) {
        $dadphase= "0|0";
        if (($kid=~/1/) && ($parentalCall=~/A|C/))
        {
                $momphase= "1|0";
        }
        elsif (($kid=~/1/) && ($parentalCall=~/G|T/))
        {
                $momphase= "0|1";
        }
        else {
                $momphase= "./.";
        }

} elsif ($mot=~/0\/1/ && $fat=~/1\/1/) {
        $dadphase= "1|1";
        if (($kid=~/0/) && ($parentalCall=~/A|C/))
        {
                $momphase= "0|1";
        }
        elsif (($kid=~/0/) && ($parentalCall=~/G|T/))
        {
                $momphase= "1|0";
        }
        else {
                $momphase= "./.";
        }
} elsif ($mot=~/0\/0/ && $fat=~/0\/0/) {
        $momphase= "0|0";
        $dadphase= "0|0";
} elsif ($mot=~/1\/1/ && $fat=~/1\/1/) {
        $momphase= "1|1";
        $dadphase= "1|1";
} elsif ($mot=~/0\/0/ && $fat=~/1\/1/) {
        $momphase= "0|0";
        $dadphase= "1|1";
} elsif ($mot=~/1\/1/ && $fat=~/0\/0/) {
        $momphase= "1|1";
        $dadphase= "0|0";
} elsif ($mot=~/0\/1/ && $fat=~/0\/1/) {
        $momphase= "./.";
        $dadphase= "./.";
} else {
        $momphase= "./.";
```

```
                                        $dadphase= "./.";
                                }
                                print OUT "$info[0]\t$eles[0]\t$eles[1]\t$eles[3]\t$eles[4]\t$momphase\t$dadphase\n";

                        }
                }
        }
}

sub get_position {
    my ($sample,$mom,$fat,$header)= @_;
    chomp $header;
    my @array = split /\t/, $header;
    my( $index1 )= grep { $array[$_] eq $sample } 0..$#array;
    my( $index2 )= grep { $array[$_] eq $mom } 0..$#array;
    my( $index3 )= grep { $array[$_] eq $fat } 0..$#array;
    return ($index1,$index2,$index3);
}

sub usage{
     print <<DIE;
     perl *.pl <chromosome> <family>
DIE
     exit 1;
}

#IN1  is the parent_hash_table.txt like this:
#progeny #parent1 #parent2 #cross_type
#A1099     JRIAL8_23           JRIAL8_23           self
#E0496     JRIAL8_23           JRIAL8_23           self
#E0927     JRIAL8_23           JRIAL8_23           self
#E0965     JRIAL8_23           JRIAL8_23           self
#JRIAL7C  JRIAL2L_index12 JRIAL2C_index3    outcross

#IN2  is bcf file w ith both parents and progeny.

#IN3  is just one header row of IN2.

#IN4  is the haplotype for each chromosome each progeny.
#A0352     1           2106411 130775647           G
#A0352     1           140850310           282167332           A
#A0352     1           283569741           306543280           G
################################################################################# code end
```
